# Sustainable and Circular Materials for Photovoltaic Power Plants: A Comparative Life Cycle Assessment of Mono-Crystalline Silicon and Perovskite Module Scenarios

**DOI:** 10.3390/ma19142996

**Published:** 2026-07-11

**Authors:** Izabela Piasecka, Patrycja Bałdowska-Witos, Patryk Leda, Grzegorz Szala, Przemysław Kubiak, Anna Leda

**Affiliations:** 1 Faculty of Mechanical Engineering, Bydgoszcz University of Science and Technology, Al. Prof. S. Kaliskiego 7, 85-796 Bydgoszcz, Poland; patrycja.baldowska-witos@pbs.edu.pl; 2 Faculty of Mechatronics, Faculty of Mechanical Engineering, Kazimierz Wielki University, Mikołaja Kopernika 1, 85-074 Bydgoszcz, Poland; patryk.leda@ukw.edu.pl (P.L.); gszala@ukw.edu.pl (G.S.); 3 Institute of Marketing and Sustainable Development, Faculty of Organization and Management, Lodz University of Technology, Al. Politechniki 8, 93-590 Łódź, Poland; przemyslaw.kubiak@p.lodz.pl; 4 Faculty of Political Science and Administration, Kazimierz Wielki University, J. Poniatowskiego 12, 85-671 Bydgoszcz, Poland; anna.leda1@ukw.edu.pl

**Keywords:** sustainable materials, circular materials, photovoltaic power plant, life cycle assessment, mono-crystalline silicon, perovskite photovoltaics, end-of-life management, recycling, resource efficiency, carbon footprint

## Abstract

Sustainable and circular materials for renewable energy applications are essential for reducing the life-cycle burdens of photovoltaic (PV) power plants and improving the resource efficiency of low-carbon energy infrastructure. This study assesses the material-related environmental performance of an existing 2 MW mono-crystalline silicon (sc-Si) photovoltaic power plant in northern Poland and a prospective perovskite solar cell (PSC) module scenario modelled as an equivalent system with the same location, installed capacity, and annual electricity output. The functional unit was defined as 2000 MWh of electricity delivered annually. A cradle-to-grave life cycle assessment (LCA) was performed in SimaPro 9.4.0 using the ReCiPe 2016 method, complemented by an Intergovernmental Panel on Climate Change (IPCC)-based greenhouse gas assessment. The inventory included photovoltaic modules, support structures, electrical installations, inverter stations, and transformers, with landfill and recycling-oriented material recovery considered as alternative post-consumer management strategies for materials after the end of the technical facility’s life. The results show that material-intensive upstream production stages and key balance-of-system components are major contributors to life-cycle impacts, while recycling can reduce selected burdens through material recovery and avoided production of primary materials. These recycling benefits were modelled using material-specific recovery rates and avoided-production credits assigned only to recovered fractions assumed to meet secondary material quality requirements. Under the adopted modelling assumptions, the PSC module scenario indicates potential for lower life-cycle impacts than the sc-Si baseline. For the prospective perovskite module scenario, this potential benefit is conditional on intact encapsulation during operation and controlled collection, separation, and recovery of lead-containing fractions at the end of life. The study demonstrates that material composition, component design, and circular end-of-life management are decisive factors for improving the environmental performance of PV power plants.

## 1. Introduction

### 1.1. Background

**Figure 1:**
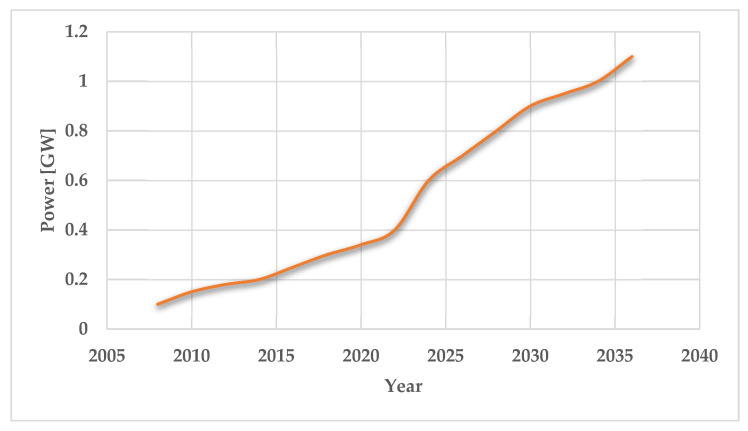
Global growth of installed photovoltaic capacity and projected expansion of solar PV as a key renewable electricity technology. Original work based on [1,2,3].

Photovoltaics (PV) is one of the fastest-growing renewable energy technologies and an important element of strategies aimed at reducing greenhouse gas emissions and increasing the share of low-carbon electricity. Global PV deployment has accelerated due to decreasing module costs, improved conversion efficiency, supportive policies, and the expansion of utility-scale and distributed systems [1,2,3]. This development confirms the role of PV in the energy transition, but also raises challenges related to material intensity, component production, and future management of post-consumer photovoltaic materials (Figure 1).

From the perspective of sustainable materials, a photovoltaic power plant is not only an electricity-generating installation, but also a complex material system composed of modules, solar glass, aluminium frames, steel structures, copper conductors, polymer encapsulants, junction boxes, inverters, transformers, cabling, and auxiliary electrical infrastructure [4,5,6]. These components differ in mass share, function, durability, recyclability, and environmental relevance. Therefore, the sustainability of PV systems not only depends on electricity output, but also on material selection, manufacturing processes, component design, service life, and recovery potential after the end of the facility’s life [7,8,9].

Crystalline silicon technologies, including mono-crystalline silicon (sc-Si), remain the dominant commercial solution because of their maturity, reliability, efficiency, and well-developed production chains [10,11,12]. At the same time, perovskite solar cell (PSC) technologies are attracting attention due to their potential for high efficiency, low-temperature processing, lightweight structures, and new applications [13,14,15]. However, their environmental performance should not only be assessed through expected technological advantages but also in relation to material composition, durability, scalability, toxicity-related issues, and end-of-life management.

The rapid expansion of PV will increase the volume of end-of-life modules and balance-of-system components. Although PV systems generate low-emission electricity during operation, production and post-consumer management may cause burdens related to energy use, resource depletion, emissions, waste generation, and land use [16,17,18]. Consequently, recycling, reuse, material recovery, and avoided production of primary raw materials are becoming important in PV environmental assessment, especially for aluminium, glass, copper, steel, silicon, silver, and selected critical or hazardous substances [19,20,21,22].

Life cycle assessment (LCA) provides a framework for evaluating these impacts across raw material extraction, component manufacturing, transport, installation, operation, maintenance, dismantling, and post-consumer material management [23,24,25]. For photovoltaic power plants, LCA enables modules and balance-of-system components to be analysed within one consistent system boundary and supports the identification of priority materials, components, and life-cycle stages for reducing impacts and improving resource efficiency [26,27].

In this context, the present study treats PV power plants as material-intensive renewable energy systems and compares an existing sc-Si installation with a prospective PSC-module scenario under equivalent functional assumptions.

### 1.2. Literature Review

Life cycle assessment (LCA) is widely used to evaluate the environmental performance of photovoltaic technologies. Early PV-related LCA studies focused mainly on greenhouse gas emissions, energy payback time, cumulative energy demand, and the benefits of replacing fossil-based electricity with solar electricity [8,11,28,29]. These studies established an important methodological basis and showed that, although PV systems generate low-emission electricity during operation, module production, auxiliary components, and material inputs may significantly affect their life-cycle profile.

Much of the literature still focuses on photovoltaic modules as the main object of analysis. Reviews by Gerbinet et al. [4], Peng et al. [5], Muteri et al. [6], and Ludin et al. [30] indicate that most studies assess module technologies, cell materials, energy payback time, greenhouse gas emissions, and selected environmental indicators. Similar trends are observed for crystalline silicon, thin-film, organic, flexible, and perovskite photovoltaic technologies [12,13,14,15,31,32,33]. Although such studies are valuable, a module-oriented approach may not fully capture the environmental consequences of utility-scale PV deployment.

Several authors have therefore emphasised the importance of balance-of-system components, including support structures, inverters, transformers, electrical installations, cabling, foundations, and auxiliary infrastructure [7,9,25,34,35]. This is particularly relevant for large-scale power plants, where steel, aluminium, copper, concrete, plastics, and electronic components are used in substantial quantities. Recent plant-level studies have assessed complete PV systems in terms of greenhouse gas emissions, energy payback periods, land-related impacts, and resource use [36,37,38,39,40,41], while broader renewable energy studies have compared solar, wind, hybrid, and region-specific electricity-generation options [27,42,43,44,45,46].

The interpretation of PV-related results also depends on the adopted life cycle impact assessment method. Many studies focus mainly on global warming potential or energy indicators, whereas material-intensive renewable energy infrastructure also requires consideration of resource depletion, toxicity, land occupation, water use, and possible burden shifting. Methods such as ReCiPe 2016 allow these aspects to be analysed through multiple midpoint categories and endpoint areas, including human health, ecosystem quality, and resource scarcity [26,47,48].

The end-of-service stage is increasingly important because of the expected growth of post-consumer photovoltaic waste. Studies on crystalline silicon modules and PV recycling show that recovery of glass, aluminium, copper, silicon, silver, and other valuable materials can reduce environmental burdens by avoiding primary material production [16,17,18,19,20,21,49,50,51]. However, these benefits depend on recovery efficiency, secondary material quality, energy demand, transport assumptions, and allocation of avoided burdens. Therefore, landfill disposal and recycling-oriented material recovery should be considered as distinct post-consumer management pathways.

Emerging photovoltaic technologies, especially perovskite solar cells, may offer environmental advantages related to lower-temperature processing, reduced material requirements, and high expected conversion efficiency [13,14,15,22,23,31,52,53]. At the same time, their assessment remains uncertain because of limited industrial maturity, durability issues, scale-up assumptions, solvent use, lead-related concerns, and the need for effective recycling and encapsulation strategies [22,23,24,52,53]. For this reason, perovskite-based systems should be interpreted in LCA as prospective or scenario-based technological options rather than as fully mature commercial equivalents of crystalline silicon PV power plants.

Overall, previous research on renewable energy systems, material depletion, ecosystem quality, and PV component management confirms that environmental performance not only depends on electricity generation but also on material composition, component structure, life-cycle stage, and post-consumer management [43,44,45,54,55,56,57,58]. Nevertheless, several gaps remain, including the continued focus on modules rather than complete power plants, the frequent prioritisation of climate and energy indicators over broader impact categories, the limited integration of end-of-life management with detailed plant-level inventories, and the rare analysis of prospective technologies such as perovskite modules within real utility-scale infrastructure. In response, this study adopts a material-oriented, plant-level LCA perspective for a real 2 MW mono-crystalline silicon photovoltaic power plant and a prospective perovskite module scenario, applying ReCiPe 2016 and IPCC methods and comparing landfill disposal with recycling-oriented material recovery.

### 1.3. Research Contribution

This study contributes to the environmental assessment of photovoltaic technologies by applying a material-oriented, plant-level life cycle assessment to a utility-scale photovoltaic power plant. Unlike module-focused studies, the analysed installation is treated as a complete material system, including photovoltaic modules, support structures, electrical installations, inverter stations, transformers, and auxiliary components. This enables the identification of the material and component groups that are most relevant for impact reduction, resource efficiency, eco-design, and recovery-oriented management.

The study develops a detailed plant-level inventory for an existing 2 MW mono-crystalline silicon photovoltaic power plant located in northern Poland and compares it with a prospective perovskite module scenario under equivalent functional assumptions. The assessment combines ReCiPe 2016 with an IPCC-based greenhouse gas evaluation, allowing both broad environmental impacts and climate-related effects to be interpreted consistently.

An additional contribution is the integration of two post-consumer material management pathways: landfill disposal and recycling-oriented material recovery. This makes it possible to evaluate the environmental role of material recovery, avoided primary material production, and recovery-related burdens within one plant-level framework. Overall, the main objective of this work is to evaluate sustainable and circular materials for photovoltaic power plants through a comparative life cycle assessment of mono-crystalline silicon and perovskite module scenarios.

## 2. Materials and Methods

### 2.1. Sustainable and Circular Materials for Photovoltaic Power Plants

In this study, a photovoltaic power plant is not only treated as an energy-generating installation, but also as a complex material system composed of functional, structural, conductive, protective, and electronic components. From this perspective, the environmental performance of the analysed facility not only depends on electricity production during operation but also on the type, quantity, origin, processing intensity, durability, and post-consumer management of the materials used in its construction [7,25,54,55,56,57,58]. This approach is consistent with the scope of sustainable materials for renewable energy applications, where particular attention is given to materials with reduced environmental burdens, recycled materials, and innovative management after the end of the technical facility’s life [16,17,18,19,20,21,49,50,51].

The analysed photovoltaic infrastructure includes both photovoltaic modules and balance-of-system components. The photovoltaic modules contain materials responsible for energy conversion, protection, encapsulation, electrical connection, and mechanical stability, including semiconductor materials, solar glass, aluminium frames, encapsulants, backsheets, junction boxes, and metallic contacts. The balance-of-system components include support structures, cabling, inverter stations, transformers, electrical installations, and auxiliary technical elements [7,25,34,54,55,56,57,58]. Although these components do not directly convert solar radiation into electricity, they are necessary for the operation of the power plant and may significantly contribute to its total material demand and environmental profile.

Sustainable materials in photovoltaic power plants can be understood as materials that enable low-emission electricity generation while reducing environmental burdens associated with extraction, processing, manufacturing, use, dismantling, and post-consumer management. In this context, material sustainability is related to several factors: lower embodied energy, reduced greenhouse gas emissions, limited use of critical or hazardous substances, durability under operational conditions, potential for repair or replacement, and suitability for recovery after the end of the technical facility’s life [21,47,59]. Therefore, the assessment of photovoltaic materials should not be limited to their functional role in energy conversion, but should also include their contribution to resource use, environmental impacts, and recovery potential.

The concept of circular materials is particularly relevant for photovoltaic infrastructure because large quantities of glass, aluminium, steel, copper, silicon, polymers, and electronic components are embedded in utility-scale installations. Circularity in this context refers to the possibility of retaining material value after use through dismantling, separation, recycling, and reintegration of secondary raw materials into production systems [49,50,51,60]. The recovery of such materials may reduce the demand for primary raw materials and avoid some environmental burdens associated with virgin material production. However, the actual environmental benefits depend on the efficiency of recovery processes, the quality of recovered materials, the energy demand of recycling operations, and the allocation approach adopted in the life cycle model. Therefore, in the present study, environmental credits were not assigned to the total mass of post-consumer materials, but only to those fractions for which separation, recovery, and substitution of primary materials were assumed to be technically feasible [16,20,50,51].

For this reason, the present study distinguishes two post-consumer material management pathways: landfill disposal and recycling-oriented material recovery. Landfill disposal represents a pathway in which materials and components are removed from the technical cycle after the end of the facility’s life and are not returned to material production chains. Recycling-oriented material recovery represents a pathway in which selected material fractions are recovered and may substitute primary materials [16,17,18,19,20,21,49,50,51,61]. This distinction enables the analysis to not only evaluate the environmental burdens generated during the life cycle of the photovoltaic power plant but also the potential role of material recovery in reducing impacts and improving resource efficiency.

The methodological approach adopted in this study, therefore, links sustainable materials, circular material management, and life cycle assessment. By analysing the photovoltaic power plant at the component and material levels, the study makes it possible to identify which parts of the installation are most relevant from the perspective of environmental impact reduction and recovery-oriented design [59,62,63]. This provides a basis for comparing mono-crystalline silicon and prospective PSC module scenarios as material configurations of photovoltaic infrastructure rather than only as energy-generation technologies.

### 2.2. Life Cycle Inventory (LCI)

Life cycle inventory (LCI) was used to quantify the input and output flows required to represent the analysed photovoltaic power plant within the adopted system boundaries, in accordance with the LCA framework [64,65]. In this study, the LCI described the installation as a material-intensive technical facility composed of photovoltaic modules, support structures, electrical installations, inverter stations, transformers, and auxiliary components.

The inventory included primary and auxiliary materials, water, fuels, electricity, emissions, waste streams, and useful electricity generation. The main life-cycle stages covered are material production, component manufacturing, construction and installation, operation, maintenance, dismantling, and post-consumer material management. Particular attention was given to material flows embedded in the photovoltaic field and balance-of-system components because these flows determine both the environmental profile of the installation and its recovery potential after the end of the technical facility’s life [7,25,54,55,56,57,58].

The LCI was structured at the component level in order to compare the mono-crystalline silicon baseline with the prospective perovskite module scenario under equivalent functional assumptions. The post-consumer stage was represented by two material management pathways: landfill disposal and recycling-oriented material recovery. This structure made it possible to account for burdens associated with material processing and recovery, as well as environmental credits related to the avoided production of virgin materials [16,17,18,19,20,21,49,50,51,62,63].

Inventory data were developed from technical documentation of the analysed photovoltaic installation, information obtained from component producers and investors, the literature data, and life cycle inventory databases. Background processes for material and energy production were represented using recognised LCI datasets, while the model structure was checked through mass and energy balance assumptions [66,67]. The resulting inventory provided the quantitative basis for assessing the environmental implications of the mono-crystalline silicon and prospective perovskite module photovoltaic power plant configurations.

### 2.3. Life Cycle Assessment (LCA)

**Figure 2:**
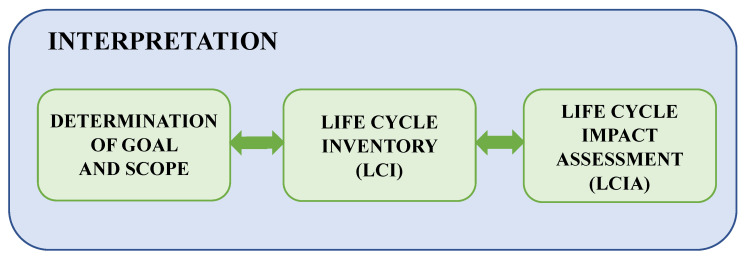
Main phases of the life cycle assessment framework according to ISO 14040 and ISO 14044: goal and scope definition, life cycle inventory analysis, life cycle impact assessment, and interpretation. Own elaboration based on [64,65].

Life cycle assessment (LCA) was applied to evaluate the environmental performance of the analysed photovoltaic power plant materials and components in accordance with the ISO 14040 and ISO 14044 framework [64,65]. The LCA procedure included goal and scope definitions, life cycle inventory analysis, a life cycle impact assessment, and an interpretation, as shown in Figure 2.

The study adopted a material-oriented, cradle-to-grave perspective, in which the photovoltaic installation was treated as a technical facility composed of photovoltaic modules, support structures, electrical installations, inverter stations, transformers, and auxiliary elements. The goal of the LCA was to compare two photovoltaic power plant configurations: the existing mono-crystalline silicon system and a prospective perovskite module scenario modelled under equivalent functional assumptions. The functional unit was linked to the annual electricity output of the analysed installation, defined as 2000 MWh of electricity delivered annually. The same system boundaries were applied to both configurations to ensure methodological consistency. 

The system boundary included raw material supply, component production, construction and installation, operation, maintenance, dismantling, and post-consumer material management. Particular attention was paid to material flows embedded in the photovoltaic field and balance-of-system components because these elements may strongly affect the environmental profile of utility-scale photovoltaic infrastructure [7,25,54,55,56,57,58]. Background processes for material and energy production were represented using recognised life cycle inventory datasets [66,67].

The post-consumer stage was modelled using two material management pathways: landfill disposal and recycling-oriented material recovery. Landfill disposal represented the removal of materials from further technical use, whereas recycling-oriented material recovery included the recovery of selected material fractions and their potential substitution for primary materials [16,17,18,19,20,21,49,50,51,62,63].

The life cycle impact assessment was performed using the ReCiPe 2016 method and complemented by an IPCC-based greenhouse gas assessment. This combination enabled the results to be interpreted both in terms of climate-related impacts and broader environmental categories related to human health, ecosystem quality, and resource scarcity [26,47,48]. The interpretation phase was used to identify dominant components, materials, life-cycle stages, and the influence of post-consumer material management on the environmental profile of the analysed photovoltaic configurations.

### 2.4. Life Cycle Impact Assessment (LCIA)

**Figure 3:**
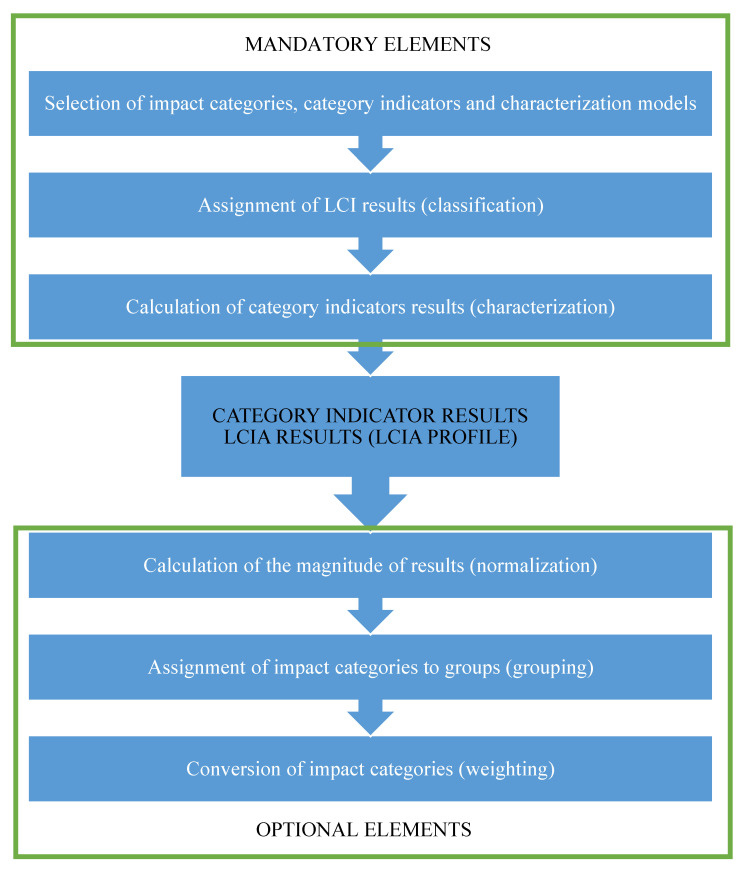
Mandatory and optional elements of the life cycle impact assessment phase according to ISO 14040 and ISO 14044. Own elaboration based on [64,65].

Life cycle impact assessment (LCIA) was used to translate the life cycle inventory results into potential environmental impacts of the analysed photovoltaic power plant materials and components, in accordance with ISO 14040 and ISO 14044 [64,65]. The LCIA procedure included the selection of impact categories, category indicators, and characterisation models, followed by classification and characterisation of inventory flows. Normalisation, grouping, and weighting were also applied where required for interpretation, as shown in Figure 3.

The assessment was performed using SimaPro 9.4.0 software and the ReCiPe 2016 method. ReCiPe 2016 was selected because it enables the evaluation of environmental impacts at both midpoint and endpoint levels [47,68]. The midpoint perspective supported the analysis of specific impact mechanisms, while the endpoint perspective aggregated results into three areas of protection: human health, ecosystem quality, and resources. This structure was suitable for assessing photovoltaic infrastructure because material production, electricity use, metal demand, land occupation, emissions, and post-consumer material management may affect different environmental areas.

In addition to the ReCiPe 2016-based assessment, an IPCC-based greenhouse gas assessment was applied to evaluate climate-related impacts. This complementary approach enabled the results to be interpreted both in terms of global warming potential and broader environmental effects related to resource use, ecosystem quality, human health, toxicity-related categories, and material recovery or loss after the end of the technical facility’s life.

The LCIA results were used to compare the mono-crystalline silicon photovoltaic power plant with the prospective perovskite module scenario under equivalent functional assumptions. They also enabled the comparison of two post-consumer material management pathways: landfill disposal and recycling-oriented material recovery. This made it possible to evaluate whether material recovery reduces environmental burdens, which impact categories are most affected by recovery processes, and how environmental credits from avoided primary material production influence the final profile of the analysed systems.

The interpretation was carried out at the level of impact categories, endpoint areas, and total environmental impact. Particular attention was paid to dominant components, materials, life-cycle stages, and possible burden shifting between impact categories. In this way, LCIA supported a material-oriented interpretation of photovoltaic power plant sustainability.

### 2.5. ReCiPe 2016

The ReCiPe 2016 method was used as the main life cycle impact assessment method for evaluating the environmental profile of the analysed photovoltaic power plant configurations. ReCiPe 2016 converts life cycle inventory results into environmental impact indicators at both midpoint and endpoint levels [47]. This structure enabled the results to be interpreted through specific environmental mechanisms and aggregated areas of protection.

At the midpoint level, ReCiPe 2016 allowed the assessment of impact categories related to climate change, resource use, toxicity, acidification, eutrophication, particulate matter formation, land use, water consumption, and other environmental mechanisms. At the endpoint level, the method aggregated midpoint results into three areas of protection: human health, ecosystem quality, and resources [47]. This approach was useful for identifying how material production, component manufacturing, electricity use, emissions, and post-consumer material management influenced the environmental profile of the analysed photovoltaic systems.

In this study, ReCiPe 2016 was applied using SimaPro 9.4.0 software. The analysis included characterisation, normalisation, grouping, and weighting, which enabled the comparison of the mono-crystalline silicon photovoltaic power plant and the prospective perovskite module scenario under equivalent functional assumptions [48,69]. The same approach was used to compare landfill disposal with recycling-oriented material recovery.

The use of ReCiPe 2016 was justified by the need to assess photovoltaic infrastructure beyond a single climate-related indicator. Although carbon footprint is important for renewable energy systems, photovoltaic power plants are also associated with material demand, metal depletion, land occupation, toxicity-related impacts, and burdens connected with component production and post-consumer treatment. Therefore, the ReCiPe 2016 results were interpreted at the level of individual impact categories, endpoint areas, and total environmental impact, with particular attention to possible burden shifting between landfill disposal and recycling-oriented material recovery.

### 2.6. IPCC

In addition to the ReCiPe 2016-based life cycle impact assessment, an IPCC-based greenhouse gas assessment was applied to evaluate the climate-related impacts of the analysed photovoltaic power plant configurations. The Intergovernmental Panel on Climate Change (IPCC) provides internationally recognised characterisation factors based on the global warming potential (GWP) metric, which allows greenhouse gas emissions from different life-cycle stages to be expressed in kg CO_2_ equivalent [70,71,72].

In this study, the IPCC-based assessment was used to calculate the carbon footprint of the analysed systems over the full life cycle, including raw material supply, component manufacturing, construction and installation, operation, maintenance, dismantling, and post-consumer material management. Particular attention was paid to emissions associated with material-intensive components, including photovoltaic modules, support structures, electrical infrastructure, inverter stations, and transformers.

The IPCC results complemented the ReCiPe 2016 assessment by providing a dedicated climate-change indicator. This enabled the mono-crystalline silicon baseline and the prospective perovskite module scenario to be compared under equivalent functional assumptions, while also assessing the influence of landfill disposal and recycling-oriented material recovery on greenhouse gas emissions.

Recycling-oriented material recovery was considered in terms of its potential to reduce greenhouse gas emissions by substituting selected primary materials with recovered secondary raw materials. The IPCC-based results were interpreted together with the ReCiPe 2016 results, so that the environmental assessment was not limited to climate change alone but also considered resource use, ecosystem quality, human health, toxicity-related impacts, and post-consumer material management. 

### 2.7. Object of Analysis

The object of analysis was an existing 2 MW mono-crystalline silicon photovoltaic power plant located in northern Poland. According to operational data provided by the investor for an eight-year period, the installation generates approximately 1900–2200 MWh of electricity per year. Due to year-to-year variability in solar irradiation and weather conditions, annual electricity production may differ by approximately 10%. Therefore, a reference annual electricity output of 2000 MWh was adopted as the functional unit for the comparative life cycle assessment. The adopted reference output corresponds to a specific yield of approximately 1000 kWh/kWp/year and a capacity factor of approximately 11.4%, which is consistent with the measured operating range of the analysed plant and typical photovoltaic yields reported for Polish conditions.

The existing mono-crystalline silicon installation was treated as the baseline configuration. In addition, a prospective perovskite module scenario was developed as a conceptual material and technological variant of the same photovoltaic facility. This scenario was modelled under equivalent functional assumptions, including the same location, installed capacity, and reference annual electricity output.

To improve the transparency of the prospective PSC scenario, the main scenario assumptions were explicitly defined. The PSC configuration was treated as a module-substitution variant of the same photovoltaic power plant, while the location, installed capacity, functional unit, reference annual electricity output, system boundary, and balance-of-system inventory were kept consistent with the sc-Si baseline. For comparability, the same 25-year plant-level service-life framework was assumed. No separate quantitative degradation curve for PSC modules was introduced because the scenario was normalised to the same reference annual electricity output. The prospective PSC inventory reflected literature-based assumptions concerning module material composition, lower material intensity, and manufacturing requirements rather than data from a mature commercial production chain. The end-of-life treatment of PSC modules followed the recycling assumptions described in Section 2.8 and the lead-related boundary conditions described in Section 2.9.

The analysed photovoltaic power plant is a material-intensive technical facility composed of photovoltaic modules, support structures, electrical infrastructure, inverter station components, transformer equipment, and auxiliary elements. The total mass of materials and components included in the analysis was approximately 300,000 kg. From the perspective of the life cycle inventory, the installation was therefore not only described as an electricity-generating system but also as a structured material system composed of functional, structural, conductive, protective, and electronic components.

The support structure of the photovoltaic modules was designed as a double-support system. This solution was required due to the specific ground conditions at the site, which was located on reclaimed landfill. The photovoltaic modules were installed with southern exposure and an inclination angle of 40°. The original installation consisted of 8334 mono-crystalline silicon photovoltaic modules, each with a rated power of 240 W. The selected module model was characterised by a maximum efficiency of 17.7%. According to the technical data provided by the investor, the manufacturer guaranteed 91.2% of the rated power during the first 10 years of operation and 80.7% during the following 15 years.

The inverter station included central inverters, a dry-type transformer, medium-voltage switchgear with gas- and air-insulated elements, a monitoring system, and DC connections for the photovoltaic modules. The station was delivered to the power plant site as a compact technical unit consisting of an insulated steel container and a concrete base. The foundation consisted of two concrete support beams. The double floor in the converter compartment enabled access to cabling and internal technical infrastructure. According to investor data, the total mass of the station was approximately 40,000 kg, and the manufacturer declared a service life of 25 years.

**Figure 4:**
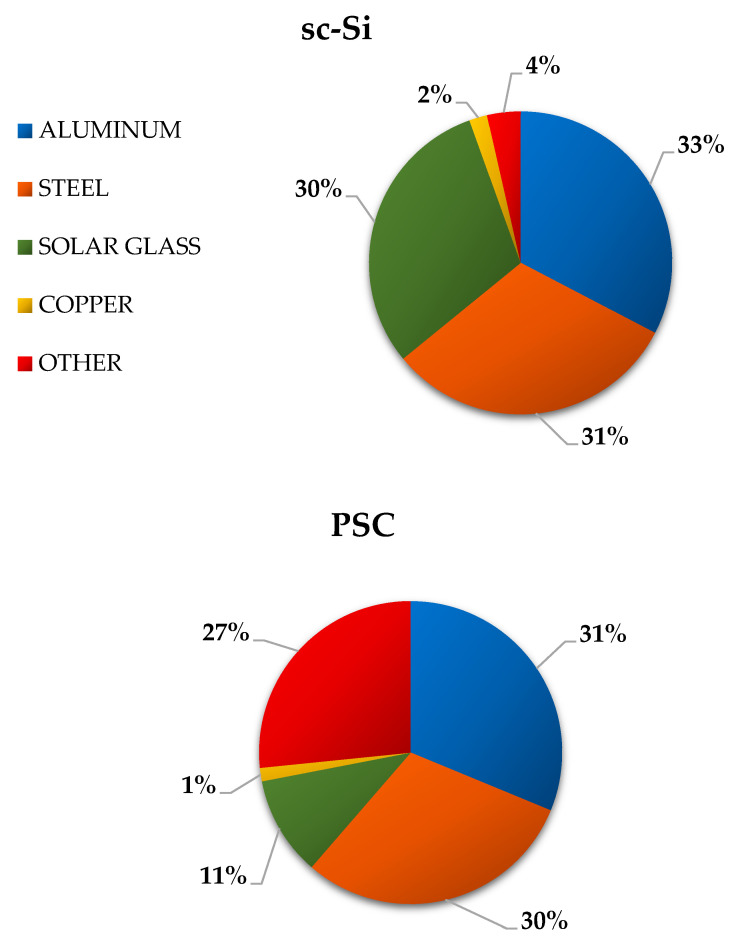
Mass shares of the main material groups included in the analysed photovoltaic power plant for the sc-Si configuration and the PSC scenario. Own elaboration based on investor data.

**Figure 5:**
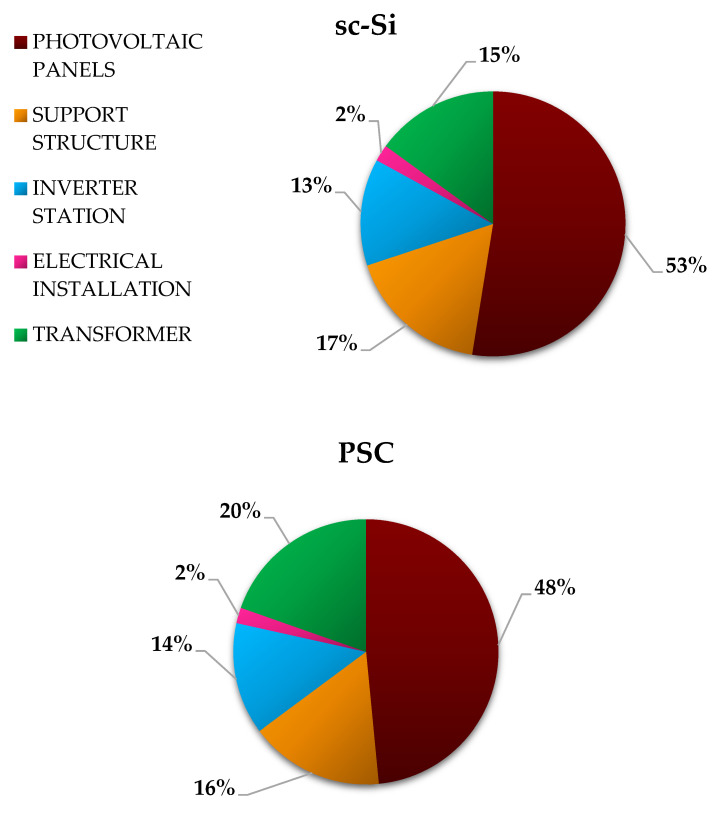
Mass shares of the main technical components included in the analysed photovoltaic power plant for the sc-Si configuration and the PSC scenario. Own elaboration based on investor data.

The material and component structure of the photovoltaic power plant was used as the basis for developing the life cycle inventory. The mass distribution of the main material groups included in the analysis is presented in Figure 4, while the mass distribution of the main technical components is shown in Figure 5. These data enabled the identification of those material fractions and system components that may significantly influence the environmental profile of the installation. Particular attention was paid to photovoltaic modules, support structures, electrical installations, inverter station elements, and the transformer because these components differ in material composition, technological function, service life, and potential for recovery after the end of the technical facility’s life.

The comparative part of the study was based on the assessment of two photovoltaic material configurations: the existing mono-crystalline silicon configuration and the prospective perovskite module scenario. The purpose of this comparison was not to describe two physically existing power plants, but to evaluate how a potential change in module technology may influence the environmental profile of a photovoltaic facility under equivalent functional and boundary assumptions. This approach made it possible to compare both configurations as material systems and to assess their performance under two post-consumer material management pathways: landfill disposal and recycling-oriented material recovery.

### 2.8. End-of-Life Recycling Model and Material Recovery Assumptions

The recycling-oriented material recovery pathway was modelled as a selective dismantling and separation scenario applied after the end of the technical facility’s life. In this pathway, the main photovoltaic power plant components were first divided into material fractions, including aluminium, steel, copper, solar glass, semiconductor-rich fractions, recoverable metals from inverter and transformer equipment, and polymeric or mixed residues. The recycling model included burdens associated with dismantling, separation, and recycling operations, as well as environmental credits related to the avoided production of primary materials.

Environmental credits were assigned only to material fractions assumed to be recovered with sufficient quality to substitute primary materials. Fractions that were not recovered, were technically difficult to separate, or were assumed to have insufficient secondary material quality were treated as residual streams and did not receive avoided-production credits. The landfill pathway was modelled as the reference post-consumer management pathway in which materials were removed from further technical circulation and no material substitution credits were assigned.

The adopted recovery rates were based on the literature data for photovoltaic module recycling, general metal recycling practice, and conservative assumptions for emerging perovskite module treatment. They should therefore be interpreted as scenario assumptions rather than guaranteed recovery efficiencies for every recycling facility [16,17,18,19,20,21,49,50,51,60,61,62,63]. The recovery-rate assumptions used in the recycling-oriented pathway are summarised in Table 1. For the PSC scenario, the semiconductor-rich and lead-containing fraction was treated as a controlled stream, with recovery or stabilisation only assumed under dedicated treatment conditions, while non-recovered lead-containing residues were assigned to controlled residual treatment.

### 2.9. Lead-Related Assumptions and Leakage-Risk Interpretation for the PSC Scenario

The prospective perovskite solar cell module scenario was additionally interpreted with respect to lead-related environmental risk because lead-containing perovskite absorber materials are one of the main environmental concerns associated with this technology [22,23,24]. The baseline LCA model did not include direct lead emissions to soil or water during normal operation. This assumption reflects an intact-module scenario in which the perovskite layer remains sealed within the module stack and protected by encapsulation during the service life of the installation.

Three lead-related situations were distinguished for interpretation. The first situation is intact operation, where no direct lead release from the module is assumed. The second situation covers accidental breakage, severe weather damage, fire exposure, or encapsulation failure, where the lead-containing layer may become exposed and create a potential pathway for contamination of water, soil, or treatment residues. The third situation covers uncontrolled disposal or improper landfilling of damaged or uncollected modules, where lead-containing fractions remain outside closed recovery systems.

The landfill pathway used in the LCA should therefore be interpreted as a comparative post-consumer material management pathway, not as a detailed site-specific leaching or contamination model. The model did not include quantitative lead-leakage factors because reliable and generally applicable industrial-scale leakage data for commercial PSC modules are still limited. Instead, lead release was treated as a risk condition that may affect the interpretation of toxicity-related impact categories.

Under the recycling-oriented material recovery pathway, the PSC scenario assumes controlled collection, separation, and treatment of lead-containing fractions. This means that the potential environmental benefits of the PSC scenario are conditional on robust encapsulation, monitoring, take-back systems, dedicated recycling processes, and appropriate management of lead-containing residues. If lead release were modelled as direct emissions to soil or water, toxicity-related categories, especially human toxicity and ecotoxicity indicators, could increase and partly offset the lower material-related burdens calculated for the prospective PSC scenario.

## 3. Results

The results of the life cycle impact assessment are presented for two photovoltaic material configurations: the existing mono-crystalline silicon photovoltaic power plant and the prospective perovskite module scenario. For each configuration, two post-consumer material management pathways were considered: landfill disposal and recycling-oriented material recovery. The results were analysed at the level of individual ReCiPe 2016 impact categories, endpoint areas, total environmental impact, and IPCC-based greenhouse gas emissions. The recycling-oriented material recovery results should be interpreted together with the recovery rates and substitution assumptions presented in Section 2.8. Negative values in selected categories do not imply that recycling processes are free of environmental burdens.

The ReCiPe 2016 results are first presented at the component level in order to identify the main contributors to the environmental profile of the analysed photovoltaic systems. The assessment includes the support structure, photovoltaic modules, inverter station, and electrical installation. This structure makes it possible to determine how different material and component groups contribute to impacts related to human health, ecosystem quality, and resource scarcity, and how these contributions change when landfill disposal is replaced by recycling-oriented material recovery.

### 3.1. ReCiPe 2016 Characterisation Results for the Mono-Crystalline Silicon Configuration

Table 2 and Table 3 present the ReCiPe 2016 characterisation results for the mono-crystalline silicon photovoltaic power plant. The results are shown separately for the main technical components and for the two post-consumer material management pathways: landfill disposal and recycling-oriented material recovery. The photovoltaic modules and the inverter station were the dominant contributors in most impact categories, while the support structure and electrical installation generally showed lower contributions.

For the mono-crystalline silicon configuration, the highest impact in the human health area was observed for the photovoltaic modules in the water consumption category under landfill disposal, reaching 3.62 × 10^1^ DALY. When recycling-oriented material recovery was applied, the same component and category showed a negative value of −1.66 × 10^1^ DALY, indicating an environmental credit associated with recovered materials and avoided primary production. A similar pattern was observed for ecosystem quality, where the highest contribution was recorded for photovoltaic modules in the water consumption, terrestrial ecosystem category, with 2.20 × 10^−1^ species/year under landfill disposal and −1.01 × 10^−1^ species/year under recycling-oriented material recovery.

In the resource-related categories, the inverter station showed the highest contribution. Under landfill disposal, fossil resource scarcity reached 6.52 × 10^4^ USD, while mineral resource scarcity reached 4.50 × 10^4^ USD. These results indicate that power-electronic and transformer-related components are particularly relevant for the resource-oriented assessment of photovoltaic infrastructure. Recycling-oriented material recovery reduced the environmental burdens in several categories, although the magnitude of the reduction differed between components and impact categories.

### 3.2. ReCiPe 2016 Characterisation Results for the Prospective Perovskite Module Scenario

Table 4 and Table 5 present the ReCiPe 2016 characterisation results for the prospective perovskite module scenario. The same component structure and post-consumer material management pathways were applied as in the mono-crystalline silicon configuration, which enabled a consistent comparison between the two photovoltaic material configurations.

For the perovskite module scenario, the highest impact in the human health area was again associated with photovoltaic modules in the water consumption category under landfill disposal, reaching 3.27 × 10^1^ DALY. Under recycling-oriented material recovery, the same category showed a negative value of −1.50 × 10^1^ DALY. In the ecosystem quality area, photovoltaic modules were also the dominant contributor in the water consumption, terrestrial ecosystem category, with 1.99 × 10^−1^ species/year under landfill disposal and −9.13 × 10^−2^ species/year under recycling-oriented material recovery.

The inverter station remained the dominant component in the resource-related categories. Under landfill disposal, fossil resource scarcity reached 5.90 × 10^4^ USD, while mineral resource scarcity reached 4.07 × 10^4^ USD. These values were lower than those obtained for the mono-crystalline silicon configuration, which indicates that the prospective perovskite module scenario may reduce selected life-cycle burdens under the adopted modelling assumptions. However, the results should be interpreted as scenario-based estimates because the perovskite configuration represents a prospective module-substitution variant rather than an existing commercial power plant.

In addition, the lower values calculated for the prospective perovskite module scenario do not include direct lead leakage from damaged modules. They should therefore be interpreted together with the lead-related assumptions described in Section 2.9. If encapsulation failure, uncontrolled disposal, or direct lead emissions to soil or water were included as explicit contamination scenarios, toxicity-related results could be higher than those obtained in the baseline PSC model.

### 3.3. Component-Level Comparison of Selected Impact Categories

**Figure 6:**
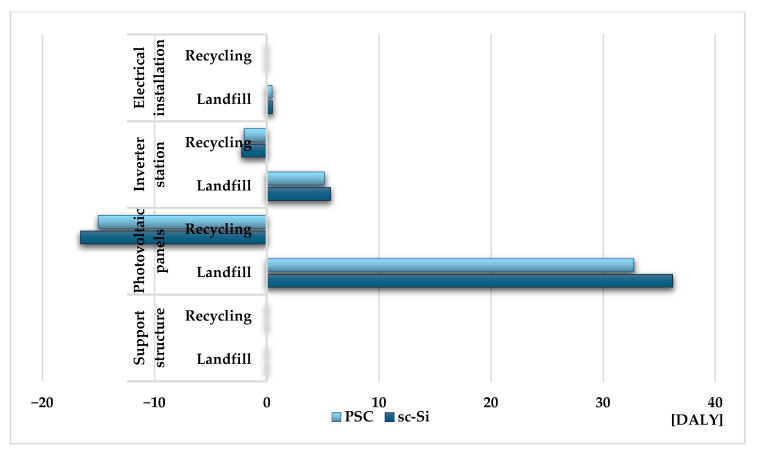
Component-level results for water consumption, human health category, by photovoltaic material configuration and post-consumer material management pathway.

**Figure 7:**
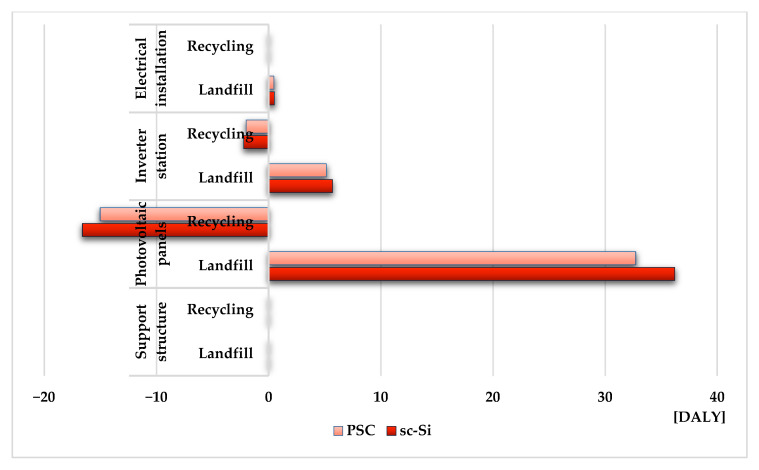
Component-level results for human non-carcinogenic toxicity by photovoltaic material configuration and post-consumer material management pathway.

**Figure 8:**
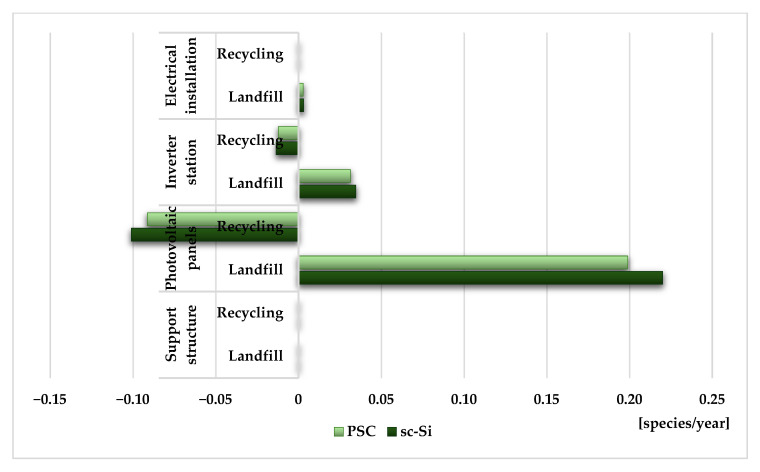
Component-level results for water consumption, terrestrial ecosystem category, by photovoltaic material configuration and post-consumer material management pathway.

**Figure 9:**
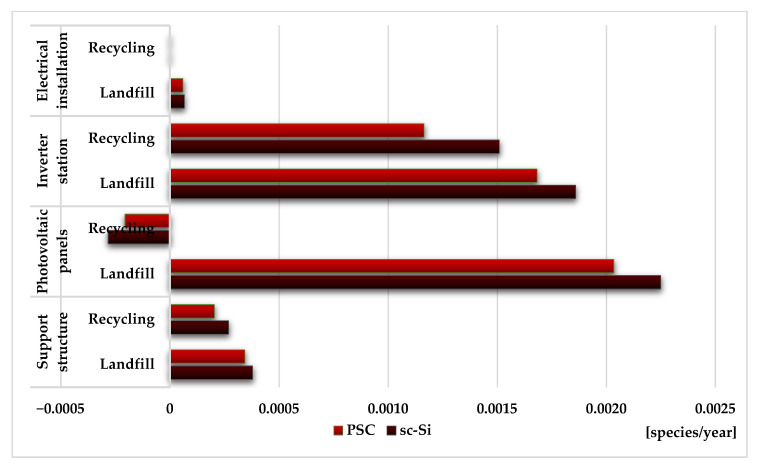
Component-level results for global warming, terrestrial ecosystems category, by photovoltaic material configuration and post-consumer material management pathway.

Table 6, Table 7, Table 8 and Table 9 and Figure 6, Figure 7, Figure 8 and Figure 9 present a component-level comparison of selected ReCiPe 2016 impact categories that were particularly relevant for the analysed photovoltaic systems. The selected categories include water consumption affecting human health, human non-carcinogenic toxicity, water consumption affecting terrestrial ecosystems, and global warming affecting terrestrial ecosystems. These categories were selected because they clearly show the environmental relevance of the main material and component groups of the photovoltaic power plant and enable the comparison of the two photovoltaic material configurations under different post-consumer material management pathways.

Table 6 and Figure 6 present the results for the water consumption, human health category. In this category, photovoltaic modules were the dominant contributors for both analysed configurations under landfill disposal. For the mono-crystalline silicon configuration, the impact of photovoltaic modules reached approximately 3.60 × 10^1^ DALY, while for the prospective perovskite module scenario, it reached approximately 3.30 × 10^1^ DALY. This indicates that, under the adopted inventory assumptions, the prospective perovskite module scenario generated a lower impact in this category. The difference can be linked mainly to the assumed material structure of the module subsystem and the lower material-related burdens attributed to the prospective perovskite module configuration.

Under recycling-oriented material recovery, the photovoltaic modules showed negative values in the water consumption, human health category for both analysed configurations, as shown in Table 6 and Figure 6. These negative values indicate environmental credits associated with the recovery of selected material fractions and the avoided production of primary materials. This result is particularly important from the perspective of sustainable and circular materials because it shows that the post-consumer management of photovoltaic modules may substantially modify the environmental profile of the entire photovoltaic facility. It also confirms that photovoltaic modules should not only be assessed in terms of electricity generation but also in terms of material composition, recovery potential, and reintegration of recovered materials into technical cycles.

Table 7 and Figure 7 show the results for the human non-carcinogenic toxicity category. In this category, the inverter station was the dominant contributor, reaching approximately 1.70 × 10^0^ DALY for the mono-crystalline silicon configuration under landfill disposal and approximately 1.60 × 10^0^ DALY for the prospective perovskite module scenario. This indicates that power-electronic, transformer-related, and auxiliary electrical components may be more relevant than photovoltaic modules for selected toxicity-related impacts. The relatively high contribution of the inverter station can be associated with the presence of metals, electronic components, insulation materials, and other technically complex material fractions that generate upstream environmental burdens.

The comparison presented in Table 7 and Figure 7 also shows that the contribution of the electrical installation was relatively low in the human non-carcinogenic toxicity category, especially under recycling-oriented material recovery. The support structure and photovoltaic modules had lower impacts than the inverter station, although the module subsystem still remained relevant. These results indicate that recovery-oriented design and post-consumer material management should not focus exclusively on photovoltaic modules. Balance-of-system components, especially inverter stations and transformer-related elements, should also be included in material recovery strategies because they may contain environmentally significant and valuable material fractions.

Table 8 and Figure 8 present the results for water consumption affecting terrestrial ecosystems. Similar to the water consumption human health category, photovoltaic modules were the dominant contributors under landfill disposal. For the mono-crystalline silicon configuration, the module-related impact reached approximately 2.20 × 10^−1^ species/year, while for the prospective perovskite module scenario, it reached approximately 2.00 × 10^−1^ species/year. This confirms that the module subsystem was the most relevant part of the installation in water consumption-related endpoint categories.

As shown in Table 8 and Figure 8, recycling-oriented material recovery substantially reduced the impacts on the water consumption terrestrial ecosystem category and resulted in negative values for photovoltaic modules. For the mono-crystalline silicon configuration, the impact decreased from approximately 2.20 × 10^−1^ species/year under landfill disposal to approximately −1.00 × 10^−1^ species/year under recycling-oriented material recovery. For the prospective perovskite module scenario, the corresponding values were approximately 2.00 × 10^−1^ species/year and −9.00 × 10^−2^ species/year. These results indicate that material recovery may play an important role in reducing ecosystem-related impacts associated with photovoltaic module materials.

Table 9 and Figure 9 show the results for global warming affecting terrestrial ecosystems. In this category, photovoltaic modules and the inverter station were the most relevant components, although the magnitude of the impacts was lower than in water consumption-related categories. Under landfill disposal, photovoltaic modules showed the highest contribution for the mono-crystalline silicon configuration, whereas the prospective perovskite module scenario showed lower module-related impacts. This confirms that the assumed material configuration of the photovoltaic module not only influences resource- and water-related indicators but also climate-related categories expressed at the ecosystem endpoint level.

The results presented in Table 9 and Figure 9 also show that recycling-oriented material recovery reduced the environmental burden in comparison with landfill disposal. The reduction was particularly visible for photovoltaic modules, where recovery of selected material fractions generated environmental credits. This confirms that the choice of post-consumer material management pathway not only influences the final waste treatment stage but also the overall life-cycle profile of the photovoltaic power plant.

The results presented in Table 6, Table 7, Table 8 and Table 9 and Figure 6, Figure 7, Figure 8 and Figure 9 demonstrate that the environmental profile of photovoltaic infrastructure depends strongly on three interrelated factors: the material composition of photovoltaic modules, the environmental burden of balance-of-system components, and the adopted post-consumer material management pathway. Photovoltaic modules were the key contributors in water consumption-related categories, whereas the inverter station was particularly relevant in the human non-carcinogenic toxicity category. At the same time, recycling-oriented material recovery consistently reduced selected impacts and, in some cases, generated environmental credits. These findings confirm the importance of analysing photovoltaic power plants as complete material systems rather than only as electricity-generating installations.

### 3.4. Endpoint Results and Total Environmental Impact

**Figure 10:**
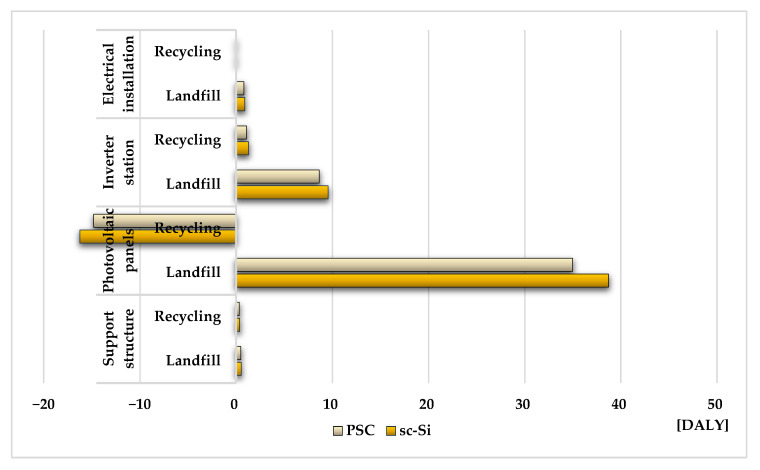
Endpoint results for human health by photovoltaic material configuration, component, and post-consumer material management pathway.

**Figure 11:**
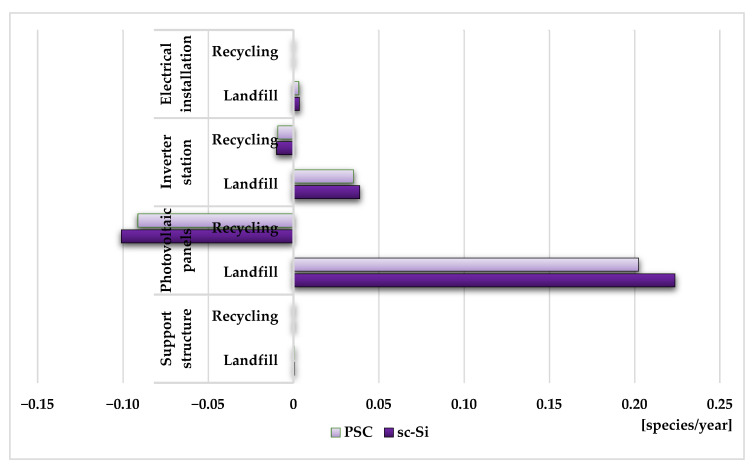
Endpoint results for ecosystems by photovoltaic material configuration, component, and post-consumer material management pathway.

**Figure 12:**
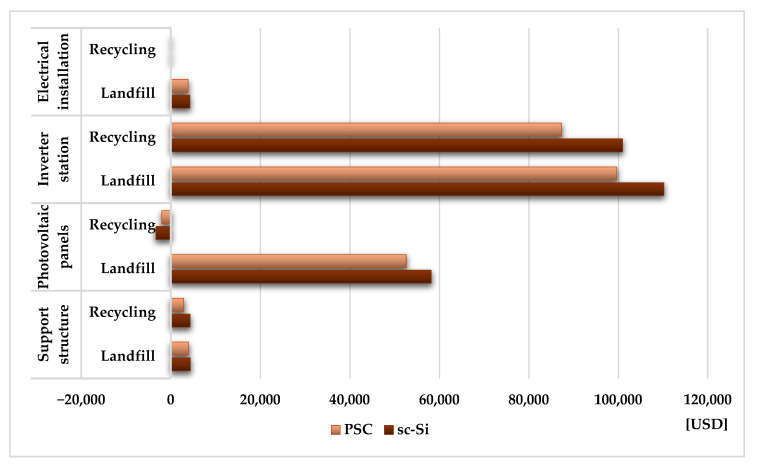
Endpoint results for resources by photovoltaic material configuration, component, and post-consumer material management pathway.

**Figure 13:**
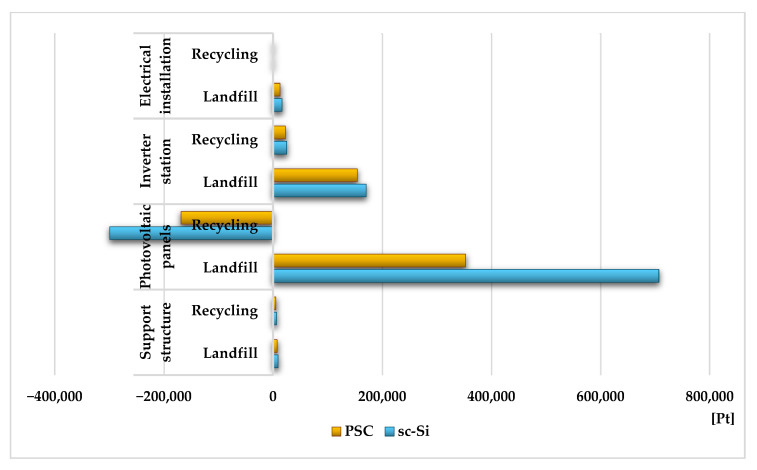
Total weighted environmental impact by component, photovoltaic material configuration, and post-consumer material management pathway.

**Figure 14:**
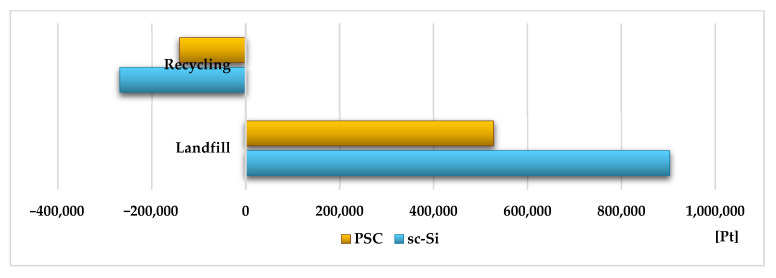
Whole-system total weighted environmental impact by photovoltaic material configuration and post-consumer material management pathway.

The endpoint results are presented in Table 10, Table 11, Table 12, Table 13, Table 14, Table 15 and Table 16 and Figure 10, Figure 11, Figure 12, Figure 13 and Figure 14. The results were aggregated into three ReCiPe 2016 areas of protection: human health, ecosystems, and resources. This level of analysis makes it possible to interpret the environmental profile of the analysed photovoltaic systems in a more integrated way than the midpoint categories alone. In particular, it enables the identification of those components and post-consumer material management pathways that are most relevant for the overall environmental performance of the mono-crystalline silicon configuration and the prospective perovskite module scenario.

Table 10 presents the endpoint results for the analysed photovoltaic power plant components in the three ReCiPe 2016 areas of protection. For both photovoltaic material configurations, the highest impacts in the human health and ecosystems areas were associated with photovoltaic modules under landfill disposal. For the mono-crystalline silicon configuration, photovoltaic modules reached 3.87 × 10^1^ DALY in the human health area and 2.24 × 10^−1^ species/year in the ecosystems area. For the prospective perovskite module scenario, the corresponding values were lower and amounted to 3.50 × 10^1^ DALY and 2.02 × 10^−1^ species/year, respectively. These results confirm that the module subsystem is the key contributor to the endpoint environmental profile in areas directly related to human health and ecosystem quality.

The results in Table 10 also show the importance of recycling-oriented material recovery. Under this pathway, the endpoint values for photovoltaic modules became negative in both the human health and ecosystems areas. For the mono-crystalline silicon configuration, the values decreased to −1.62 × 10^1^ DALY and −1.01 × 10^−1^ species/year, respectively. For the prospective perovskite module scenario, they decreased to −1.48 × 10^1^ DALY and −9.12 × 10^−2^ species/year. These negative values indicate environmental credits associated with the recovery of selected material fractions and the avoided production of primary materials. In the resources area, the highest contribution was observed for the inverter station, reaching 1.10 × 10^5^ USD for the mono-crystalline silicon configuration and 9.97 × 10^4^ USD for the prospective perovskite module scenario under landfill disposal.

Table 11 and Figure 10 present the endpoint results for the human health area. The comparison confirms that photovoltaic modules were the dominant contributors under landfill disposal. The impact of the mono-crystalline silicon photovoltaic modules reached approximately 3.90 × 10^1^ DALY, while the corresponding value for the prospective perovskite module scenario was approximately 3.50 × 10^1^ DALY. The lower value obtained for the perovskite module scenario suggests that, under the adopted inventory assumptions, the prospective module substitution may reduce selected human health-related impacts. However, this result should be interpreted as a scenario-based estimate rather than as a fully validated industrial-scale performance of perovskite photovoltaic technology.

As shown in Table 11 and Figure 10, recycling-oriented material recovery substantially changed the human health results for photovoltaic modules. Negative values were obtained for both module configurations, indicating environmental credits resulting from the recovery and reuse of materials. The inverter station was the second most important contributor in this area, although its impact was considerably lower than that of photovoltaic modules under landfill disposal. The support structure and electrical installation showed smaller contributions, especially under recycling-oriented material recovery. These results demonstrate that human health-related impacts are strongly influenced by the material intensity and recovery potential of the photovoltaic module subsystem.

Table 12 and Figure 11 show the endpoint results for the ecosystem area. The dominant role of photovoltaic modules was again visible under landfill disposal. For the mono-crystalline silicon configuration, photovoltaic modules reached approximately 2.20 × 10^−1^ species/year, while the prospective perovskite module scenario reached approximately 2.00 × 10^−1^ species/year. The inverter station was the second most relevant component, whereas the support structure and electrical installation showed lower contributions. This indicates that ecosystem-related impacts are primarily shaped by the material composition and life-cycle burdens of photovoltaic modules, followed by balance-of-system components with complex material structures.

The results presented in Table 12 and Figure 11 also indicate that recycling-oriented material recovery reduced ecosystem-related impacts and generated negative values for photovoltaic modules. This means that the recovery of selected material fractions may compensate for part of the burdens associated with material production and end-of-service treatment. The results, therefore, confirm that circular material management is particularly important for reducing ecosystem-related impacts of photovoltaic infrastructure. From the perspective of sustainable materials, the module subsystem should be treated as a priority area for recovery-oriented design, dismantling, and recycling.

Table 13 and Figure 12 present the endpoint results for the resources area. In contrast to the human health and ecosystems areas, the highest contribution was associated with the inverter station rather than photovoltaic modules. Under landfill disposal, the resource-related impact of the inverter station reached approximately 1.10 × 10^5^ USD for the mono-crystalline silicon configuration and 9.96 × 10^4^ USD for the prospective perovskite module scenario. This confirms that resource-related impacts are strongly influenced by power-electronic and transformer-related components, which contain metals, electronic parts, and other material fractions associated with high upstream resource burdens.

The results in Table 13 and Figure 12 also show that photovoltaic modules contributed substantially to the resources area, but their contribution was lower than that of the inverter station under landfill disposal. Recycling-oriented material recovery reduced resource-related impacts for several components and, in the case of photovoltaic modules, resulted in negative values. This indicates that recovered material fractions may substitute primary raw materials and reduce the pressure on resource use. However, the inverter station remained a significant contributor even under recycling-oriented material recovery, which suggests that balance-of-system components should be included in circularity strategies for photovoltaic power plants.

Table 14 presents the weighted ReCiPe 2016 results divided into human health, ecosystems, resources, and total environmental impact. The weighted results confirm the importance of photovoltaic modules in the total environmental profile of both analysed configurations. Under landfill disposal, the total impact of photovoltaic modules reached 7.07 × 10^5^ Pt for the mono-crystalline silicon configuration and 3.53 × 10^5^ Pt for the prospective perovskite module scenario. The inverter station was the second most important contributor, reaching 1.71 × 10^5^ Pt for the mono-crystalline silicon configuration and 1.54 × 10^5^ Pt for the prospective perovskite module scenario under landfill disposal.

The results in Table 14 further show that recycling-oriented material recovery substantially reduced the weighted environmental impacts. For photovoltaic modules, the total weighted values became negative and amounted to −2.99 × 10^5^ Pt for the mono-crystalline silicon configuration and −1.69 × 10^5^ Pt for the prospective perovskite module scenario. These values indicate environmental credits resulting from material recovery. At the same time, the inverter station remained an important contributor to the weighted results, especially in the resources area. This confirms that both photovoltaic modules and balance-of-system components determine the overall environmental profile of photovoltaic infrastructure.

Table 15 and Figure 13 summarise the weighted total environmental impact by component, photovoltaic module type, and post-consumer material management pathway. The comparison clearly shows that photovoltaic modules were the dominant component under landfill disposal. The mono-crystalline silicon module configuration had the highest total impact, reaching 7.07 × 10^5^ Pt, while the prospective perovskite module scenario reached 3.52 × 10^5^ Pt. The inverter station also made a significant contribution, whereas the support structure and electrical installation were less important in the total weighted result.

As shown in Table 15 and Figure 13, recycling-oriented material recovery reduced the total weighted impacts for all major components. The strongest effect was observed for photovoltaic modules, where negative values were obtained for both analysed configurations. This indicates that module recycling and material recovery can play a decisive role in reducing the total environmental burden of photovoltaic power plants. The comparison also shows that the prospective perovskite module scenario had lower total weighted impacts than the mono-crystalline silicon configuration under both post-consumer material management pathways, although this result remains dependent on the assumptions adopted for the prospective inventory model.

Table 16 and Figure 14 present the whole-system weighted results for the two photovoltaic material configurations and the two post-consumer material management pathways. Under landfill disposal, the total weighted impact amounted to 9.03 × 10^5^ Pt for the mono-crystalline silicon configuration and 5.27 × 10^5^ Pt for the prospective perovskite module scenario. This means that, under the adopted assumptions, the prospective perovskite module scenario showed a lower total environmental burden than the existing mono-crystalline silicon configuration.

Under recycling-oriented material recovery, the total weighted results decreased to −2.67 × 10^5^ Pt for the mono-crystalline silicon configuration and −1.40 × 10^5^ Pt for the prospective perovskite module scenario, as presented in Table 16 and Figure 14. The negative values indicate that the environmental credits associated with recovered materials exceeded the burdens assigned to recovery processes within the adopted modelling framework. These results highlight the importance of post-consumer material management for photovoltaic infrastructure and confirm that recycling-oriented material recovery can significantly change the final environmental profile of both analysed photovoltaic material configurations.

These credits are conditional on the assumed recovery efficiencies and on the ability of recovered fractions to substitute primary materials. If lower recovery rates, lower secondary material quality, or higher recycling energy demand were assumed, the magnitude of the negative values would decrease, and the total environmental benefit of the recycling-oriented pathway would be smaller.

The endpoint and weighted results presented in Table 10, Table 11, Table 12, Table 13, Table 14, Table 15 and Table 16 and Figure 10, Figure 11, Figure 12, Figure 13 and Figure 14 demonstrate that the environmental performance of photovoltaic power plants not only depends on the type of photovoltaic module but also on the material structure of balance-of-system components and the selected post-consumer material management pathway. Photovoltaic modules dominated the human health and ecosystems areas, whereas the inverter station was the most important contributor in the resources area. The results also confirm that recycling-oriented material recovery can generate substantial environmental credits, supporting the relevance of circular material strategies for sustainable photovoltaic infrastructure. 

### 3.5. IPCC-Based Greenhouse Gas Assessment

**Figure 15:**
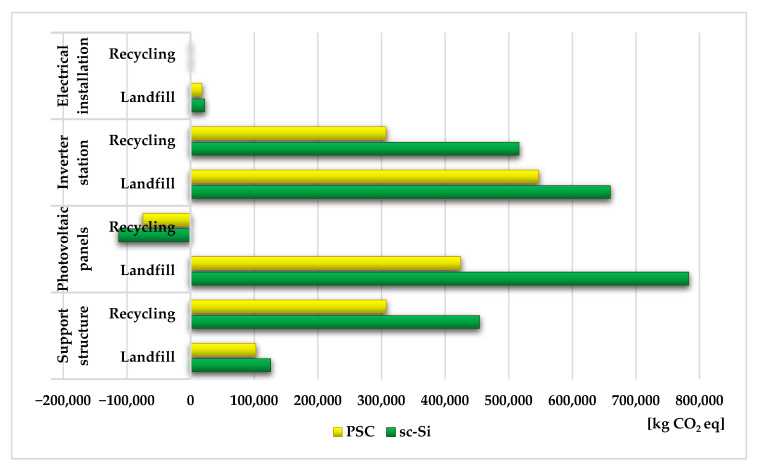
Component-level greenhouse gas emissions by photovoltaic material configuration and post-consumer material management pathway.

**Figure 16:**
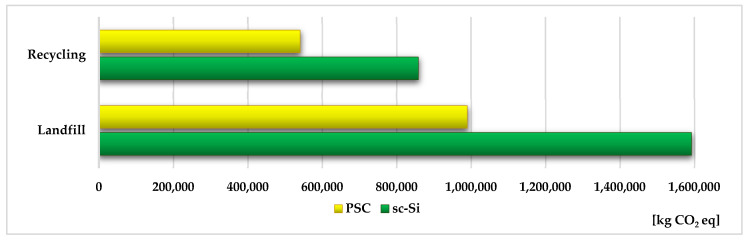
Whole-system greenhouse gas emissions by photovoltaic material configuration and post-consumer material management pathway.

The IPCC-based greenhouse gas assessment results are presented in Table 17, Table 18 and Table 19 and Figure 15 and Figure 16. The results are expressed in kg CO_2_ equivalent and include fossil, biogenic, and land transformation-related greenhouse gas emissions. This part of the analysis complements the ReCiPe 2016 results by focusing specifically on the climate-related consequences of the analysed photovoltaic material configurations. The comparison was conducted for the existing mono-crystalline silicon configuration and the prospective perovskite module scenario, taking into account two post-consumer material management pathways: landfill disposal and recycling-oriented material recovery.

Table 17 presents the detailed IPCC characterisation results for individual greenhouse gas emission groups: GWP100 fossil, GWP100 biogenic, and GWP100 land transformation. The results show that fossil greenhouse gas emissions were the dominant contributor to the total carbon footprint of the analysed photovoltaic systems. This indicates that the main climate-related burdens were associated primarily with fossil energy use in upstream processes, especially material production, component manufacturing, and the production of technically complex infrastructure elements.

For the mono-crystalline silicon configuration under landfill disposal, the highest fossil GWP100 contribution was observed for photovoltaic modules, reaching 7.34 × 10^5^ kg CO_2_ eq. The inverter station was the second most important contributor, with 6.37 × 10^5^ kg CO_2_ eq. For the prospective perovskite module scenario, the corresponding fossil GWP100 values were lower and amounted to 3.97 × 10^5^ kg CO_2_ eq for photovoltaic modules and 5.76 × 10^5^ kg CO_2_ eq for the inverter station. These results indicate that, under the adopted inventory assumptions, the prospective perovskite module scenario reduced the fossil-related climate burden of the module subsystem, while the inverter station remained a major contributor in both configurations.

Table 17 also shows that biogenic and land transformation-related greenhouse gas emissions were considerably lower than fossil greenhouse gas emissions for most components. Nevertheless, they were included in the analysis to provide a complete IPCC-based greenhouse gas profile. The relatively small contribution of the electrical installation was also visible in this table. For both photovoltaic material configurations, the electrical installation generated the lowest or one of the lowest contributions in all GWP100 emission groups, particularly under recycling-oriented material recovery.

The results presented in Table 17 further confirm the importance of recycling-oriented material recovery. For photovoltaic modules, recycling reduced fossil GWP100 values substantially and, in some cases, resulted in negative values. These negative values indicate environmental credits associated with the recovery of selected material fractions and the avoided production of primary materials. This result is particularly relevant from the perspective of sustainable and circular materials because it demonstrates that post-consumer material recovery can directly influence the carbon footprint of photovoltaic infrastructure.

Table 18 presents the summarised IPCC greenhouse gas emissions for the main components of the analysed photovoltaic systems. For the mono-crystalline silicon configuration under landfill disposal, photovoltaic modules generated 7.83 × 10^5^ kg CO_2_ eq, while the inverter station generated 6.59 × 10^5^ kg CO_2_ eq. These two components, therefore, dominated the carbon footprint of the system. The support structure contributed 1.26 × 10^5^ kg CO_2_ eq, while the electrical installation had a much lower contribution, equal to 2.24 × 10^4^ kg CO_2_ eq.

For the prospective perovskite module scenario, Table 18 shows lower greenhouse gas emissions for most components under landfill disposal. The photovoltaic modules generated 4.24 × 10^5^ kg CO_2_ eq, while the inverter station generated 5.47 × 10^5^ kg CO_2_ eq. In this configuration, the inverter station became the dominant contributor to the carbon footprint, exceeding the contribution of the photovoltaic modules. This shift is important because it indicates that, when the module-related burden is reduced in the prospective perovskite module scenario, balance-of-system components become relatively more important in the climate-related profile of the photovoltaic power plant.

Figure 15 visualises the component-level greenhouse gas emissions presented in Table 18. The figure confirms that photovoltaic modules and the inverter station are the main contributors to the carbon footprint of both analysed photovoltaic material configurations. It also clearly shows the reduction in greenhouse gas emissions obtained when landfill disposal is replaced by recycling-oriented material recovery. The reduction is particularly visible for the photovoltaic module subsystem, which indicates that module material recovery may play a key role in reducing the climate impact of photovoltaic infrastructure.

The comparison shown in Figure 15 also demonstrates that the prospective perovskite module scenario had lower component-level greenhouse gas emissions than the mono-crystalline silicon configuration under the adopted modelling assumptions. However, this result should be interpreted with caution because the perovskite configuration represents a prospective module-substitution scenario rather than a real operating power plant. Therefore, the observed reduction should be understood as the potential climate-related benefit of a prospective material configuration, not as a confirmed industrial-scale performance of perovskite photovoltaic technology.

Table 19 presents the whole-system IPCC greenhouse gas emissions for the two photovoltaic material configurations and the two post-consumer material management pathways. Under landfill disposal, the total carbon footprint of the mono-crystalline silicon configuration amounted to 1.59 × 10^6^ kg CO_2_ eq. For the prospective perovskite module scenario, the corresponding value was 9.89 × 10^5^ kg CO_2_ eq. This means that, within the adopted modelling framework, the prospective perovskite module scenario showed a lower total carbon footprint than the existing mono-crystalline silicon configuration.

Table 19 also shows that recycling-oriented material recovery reduced greenhouse gas emissions in both analysed configurations. For the mono-crystalline silicon configuration, the total carbon footprint decreased from 1.59 × 10^6^ kg CO_2_ eq under landfill disposal to 8.57 × 10^5^ kg CO_2_ eq under recycling-oriented material recovery. For the prospective perovskite module scenario, the corresponding values decreased from 9.89 × 10^5^ kg CO_2_ eq to 5.40 × 10^5^ kg CO_2_ eq. These results indicate that recycling-oriented material recovery may reduce the carbon footprint of photovoltaic infrastructure by substituting selected primary materials with recovered secondary materials.

This reduction reflects the avoided-production approach adopted in the recycling model. It is therefore sensitive to the assumed recovery rates for aluminium, steel, copper, glass, semiconductor-rich fractions, and recoverable metals from electrical equipment, as well as to the quality of the secondary materials obtained.

Figure 16 presents the whole-system greenhouse gas emissions and visually confirms the trends shown in Table 18. The mono-crystalline silicon configuration had the highest carbon footprint under landfill disposal, while the prospective perovskite module scenario showed lower greenhouse gas emissions in both post-consumer material management pathways. At the same time, both configurations benefited from recycling-oriented material recovery, which reduced total greenhouse gas emissions in comparison with landfill disposal.

The IPCC-based results presented in Table 17, Table 18 and Table 19 and Figure 15 and Figure 16 confirm that the climate-related profile of photovoltaic power plants is shaped mainly by photovoltaic modules and the inverter station. The results also show that recycling-oriented material recovery can substantially reduce greenhouse gas emissions by enabling the recovery of selected material fractions and limiting the need for primary material production. From the perspective of sustainable and circular materials, these findings highlight the importance of integrating material recovery strategies into the design, dismantling, and post-consumer management of photovoltaic infrastructure.

## 4. Discussion

### 4.1. Material-Oriented Interpretation of the Life Cycle Results

The results confirm that the environmental performance of photovoltaic power plants cannot only be interpreted through the amount of low-carbon electricity generated during operation. From the perspective of sustainable materials for renewable energy applications, a photovoltaic power plant should be considered as a complex material system composed of functional, structural, conductive, protective, and electronic components. The environmental profile of the analysed systems was strongly influenced by material composition, component mass, technological complexity, and the adopted post-consumer material management pathway [73,74,75].

The component-level results showed that photovoltaic modules and the inverter station were the main contributors to the environmental profile of the analysed systems. Photovoltaic modules dominated several human health and ecosystem-related categories, especially those associated with water consumption, whereas the inverter station was particularly important in resource-related categories and selected toxicity-related indicators. This confirms that the assessment of photovoltaic infrastructure should include both modules and balance-of-system components [7,25,34,54,55,56,57,58].

This finding is important for sustainable and circular materials research. A module-only assessment may underestimate the contribution of power-electronic and auxiliary infrastructure, while an energy-only interpretation may hide the material-related causes of environmental burdens. The plant-level, component-oriented approach adopted in this study therefore provides a basis for identifying material priorities in eco-design, recovery-oriented design, dismantling strategies, and post-consumer material management [60,62,63,75,76,77,78,79,80,81,82].

### 4.2. Role of Post-Consumer Material Management Pathways

The comparison of landfill disposal and recycling-oriented material recovery demonstrated that post-consumer material management can substantially change the environmental profile of photovoltaic infrastructure. In several categories, recycling-oriented material recovery reduced environmental burdens and generated negative values, interpreted as environmental credits associated with recovered material fractions, and avoided production of primary materials. This was particularly relevant for aluminium, steel, copper, glass, silicon-based fractions, and selected electronic components [16,17,18,19,20,21,49,50,51,76,77,78,79,80,81,82].

The recycling-oriented pathway should therefore be understood as a material recovery scenario with explicit recovery and substitution assumptions. The largest recovery rates were assigned to metallic fractions, namely aluminium frames and support elements, copper cables and conductive elements, and steel support structures. These materials have relatively well-established recycling routes and high potential to substitute primary materials. Lower recovery rates were assigned to solar glass, semiconductor-rich fractions, and complex electrical or electronic equipment because their recovery depends more strongly on separation efficiency, contamination, secondary material quality, and the availability of specialised treatment processes.

The modelling approach also distinguishes between high-value recyclable fractions and mixed residual materials. Polymeric, laminated, and composite fractions were assigned limited or no recovery credits unless they were assumed to be technically separable and reusable. This is important because photovoltaic modules and balance-of-system components contain both easily recoverable bulk materials and complex mixed fractions. As a result, the environmental benefit of recycling is not proportional to the total mass of waste, but to the mass and quality of the fractions that can actually be recovered and used as secondary raw materials.

The results, therefore, support recycling-oriented material management, but they should not be interpreted as evidence that every recycling pathway will generate the same level of benefit. The magnitude of environmental credits depends on recovery efficiency, energy demand of recycling operations, transport and logistics, allocation rules, and the ability of secondary materials to replace primary production. These conditions are especially relevant for emerging PSC modules, where semiconductor-rich and lead-containing fractions require controlled and specialised treatment.

The environmental benefits of recycling were not uniform across all components. Photovoltaic modules showed the strongest shift towards environmental credits, whereas the inverter station retained positive burdens in several categories. This indicates that recovery efficiency not only depends on the presence of valuable materials but also on material heterogeneity, component architecture, ease of dismantling, separation efficiency, and the quality of recovered fractions. Therefore, recycling should not only be treated as a final waste treatment but as a material management pathway that should already be considered at the design and planning stages [62,79,81,82].

The results also show that circularity strategies should not focus exclusively on photovoltaic modules. Inverters, transformers, switchgear, cabling, and monitoring systems include metals and electronic components that may be associated with resource depletion and material criticality. Therefore, the entire photovoltaic power plant should be considered as a recoverable material system, especially in the context of increasing demand for critical and strategic raw materials in renewable energy technologies [59,83]. The usefulness of LCA in evaluating post-use management has also been demonstrated for other technical products, where it supported the comparison of different material treatment options after use [84].

### 4.3. Implications for Sustainable and Circular Photovoltaic Materials

The obtained results have direct implications for the development of sustainable and circular photovoltaic materials. Photovoltaic modules should remain a priority area for eco-design, durability improvement, dismantling, and recovery-oriented planning because they dominate several impact categories. Their environmental relevance is related to the use of glass, aluminium frames, encapsulants, backsheets, semiconductor layers, metallic contacts, junction boxes, and other auxiliary fractions.

High-volume material fractions, such as solar glass, aluminium, steel, and copper, are particularly important because they represent a large part of the mass of photovoltaic infrastructure and may offer significant recovery potential. Their recycling can reduce demand for primary raw materials, but the environmental benefits depend on the quality, purity, and practical usability of recovered materials [60,75,76,80,81,82,83,84,85].

The results also indicate that technologically complex components require more attention. The inverter station and transformer-related elements showed high relevance in resource-related and toxicity-related categories. Therefore, sustainable material strategies for photovoltaic power plants should not only include module recycling but also recovery-oriented management of balance-of-system components. This requires modularity, clear material documentation, separability of material fractions, and reduction in material complexity.

### 4.4. Interpretation of the Prospective Perovskite Module Scenario

The prospective perovskite module scenario showed lower environmental burdens than the mono-crystalline silicon configuration under the adopted modelling assumptions. This suggests that emerging photovoltaic materials may offer environmental advantages if they are introduced into systems with lower material intensity, reduced processing burdens, and effective recovery pathways. Similar conclusions have been reported in LCA studies of perovskite photovoltaic technologies and perovskite/silicon tandem concepts [13,14,15,22,23,24,52,53,86,87,88,89,90,91,92,93,94,95].

However, this result should be interpreted with caution. The perovskite module configuration represents a prospective module-substitution scenario, not an existing commercial photovoltaic power plant. Its environmental profile depends on assumptions concerning material composition, manufacturing processes, module efficiency, durability, encapsulation, lead-related risk management, solvent use, manufacturing yield, and recycling routes.

The 25-year service-life framework should therefore be understood as a comparability assumption rather than as a confirmed industrial lifetime for commercial PSC modules. If PSC modules had a shorter service life, faster degradation, or required additional replacement during the analysed period, the environmental burden per unit of electricity would increase. Similarly, higher material losses, lower manufacturing yield, or higher energy demand during industrial-scale PSC production could reduce the apparent environmental advantage of the prospective PSC scenario.

Lead management is the most important risk-specific issue for the prospective PSC scenario. Although the mass of lead-containing absorber material is small compared with high-volume fractions such as glass, aluminium, steel, and copper, its potential toxicity makes it environmentally relevant. Therefore, the lower environmental burdens obtained for the PSC scenario should not be interpreted as unconditional environmental superiority over the mono-crystalline silicon configuration. They represent a managed scenario in which modules remain encapsulated during operation and lead-containing fractions are collected and treated in controlled end-of-life systems.

The lead-related risk differs between operational and post-consumer conditions. During intact operation, the main protective function is provided by encapsulation and the module stack, which limit direct exposure of the absorber layer. Under accidental breakage, severe mechanical damage, fire exposure, or encapsulation failure, the lead-containing layer may become accessible to water, soil, or residues generated during emergency handling and treatment. Under uncontrolled landfill disposal, the risk is related to the long-term presence of lead-containing fractions outside technical recovery loops. These conditions could increase toxicity-related impacts if direct emissions were included in the model.

For this reason, the PSC scenario should be interpreted as environmentally favourable only under strict boundary conditions. These include durable encapsulation, monitoring of module integrity, take-back obligations, separation of lead-containing layers, specialist recovery or stabilisation of lead-bearing residues, and avoidance of uncontrolled disposal. Without these conditions, the environmental advantage of the PSC module scenario could be reduced, especially in human toxicity and ecotoxicity-related categories [22,23,24,85,86,87,88,89,90,91,92,96,97].

Therefore, the results should be treated as an indication of potential environmental benefits rather than as confirmation of the industrial-scale superiority of perovskite modules. Future improvements in durability, scalable low-energy manufacturing, encapsulation, and material recovery may strengthen the environmental advantages of this technology, whereas short service life, inefficient recovery, or high manufacturing losses may reduce them [96,97,98].

### 4.5. Limitations and Future Research Directions

Several limitations should be considered. First, the mono-crystalline silicon configuration was based on an existing photovoltaic power plant, whereas the perovskite module configuration was modelled as a prospective scenario. The comparison, therefore, combines real plant-level inventory data with assumptions concerning an emerging photovoltaic material technology.

Because the comparison combines an existing sc-Si photovoltaic power plant with a prospective PSC module-substitution scenario, the final results are expected to be most sensitive to several groups of modelling parameters. The first group includes module service life, degradation behaviour, and annual electricity output because these parameters determine the environmental burden assigned to the functional electricity output. Shorter module lifetime or higher degradation would increase the burden per MWh, especially for the prospective PSC scenario. The second group includes the electricity mix and the energy demand of module and component manufacturing because upstream material and component production were among the main contributors to life-cycle impacts. The third group includes recycling rates, recovery efficiencies, secondary material quality, and avoided-production substitution ratios because these parameters determine the magnitude of environmental credits assigned to material recovery. Lower recovery efficiencies or lower-quality secondary materials would reduce the benefits of recycling-oriented material recovery. The fourth group includes PSC-specific parameters, especially manufacturing yield, encapsulation durability, recovery of semiconductor-rich fractions, and controlled management of lead-containing residues. These parameters may influence both the overall environmental profile and toxicity-related interpretation of the PSC scenario. A full quantitative sensitivity analysis was beyond the scope of the present study, but these parameters should be prioritised in future uncertainty and sensitivity analyses.

Among the modelling assumptions, the greatest influence on the final results is expected from four parameter groups. The first group includes module service life, degradation, and annual electricity output because these parameters determine the environmental burden assigned to the functional electricity output. The second group includes the electricity mix and energy demand of module and component manufacturing because upstream material production is one of the main contributors to life-cycle impacts. The third group includes recycling rates, secondary material quality, and substitution ratios because these parameters determine the magnitude of avoided-production credits. The fourth group includes PSC-specific assumptions, especially manufacturing yield, encapsulation durability, recovery of semiconductor-rich fractions, and controlled management of lead-containing residues. Transport distances and residual treatment assumptions may also influence the results, but they are expected to be less decisive than the above parameter groups within the adopted model.

An additional limitation concerns the lead-related interpretation of the prospective PSC scenario. The study did not include a quantitative fate, transport, or leaching model for lead released from damaged or improperly managed PSC modules. Direct lead emissions to soil or water were not included in the baseline LCA inventory. This was due to the limited availability of validated industrial-scale leakage factors for commercial PSC modules and the prospective character of the analysed technology. Consequently, the lead-related assessment should be understood as a risk-based interpretation rather than a full contamination scenario.

Future research should therefore include sensitivity analyses for lead release under damaged-module conditions, fire exposure, encapsulation failure, and uncontrolled disposal. Such analyses should be linked with site-specific leaching behaviour, local soil and water conditions, collection efficiency, recycling technology, and the effectiveness of lead recovery or stabilisation processes.

Second, the results depend on recovery assumptions, allocation procedures, background databases, energy mix, and the assumed ability of recovered materials to substitute primary materials. If recovered materials do not reach sufficient quality or if recycling processes require high energy inputs, the environmental credits may be lower.

An additional limitation concerns the recycling model itself. The recovery rates used in the study represent scenario assumptions based on the available literature and typical material recovery routes, rather than facility-specific operating data from one dedicated recycling plant. The model does not include detailed process parameters for individual recycling technologies, such as exact separation yields, contamination levels, losses during refining, or variability in secondary material quality. Consequently, the environmental credits should be interpreted as conditional credits that depend on the technical performance of recycling processes and on the actual market substitution of primary materials.

Third, the study focused on environmental indicators and did not include a full techno-economic assessment of recycling processes or a detailed analysis of social aspects. The practical implementation of recycling-oriented material recovery will also depend on costs, logistics, regulatory conditions, availability of recycling infrastructure, and market demand for secondary raw materials.

Future research should focus on more detailed inventory data for industrial-scale perovskite modules. Further studies should also assess recycling technologies for perovskite modules and balance-of-system components, especially in relation to recovery efficiency, secondary material quality, emissions during treatment, and the recovery of critical raw materials. Future research should therefore include sensitivity analyses for recovery rates, recycling energy demand, transport distances, and secondary material substitution ratios. Such analyses would make it possible to evaluate how robust the environmental benefits of recycling are under less favourable or more conservative end-of-life conditions [99,100].

## 5. Conclusions

This study evaluated sustainable and circular materials for photovoltaic power plants through a comparative life cycle assessment of an existing mono-crystalline silicon photovoltaic installation and a prospective perovskite module scenario. The analysis was performed at the plant and component levels and included two post-consumer material management pathways: landfill disposal and recycling-oriented material recovery.

The results showed that photovoltaic modules and the inverter station were the dominant contributors to the environmental profile of the analysed systems. Photovoltaic modules were particularly important in human health and ecosystem-related categories, while the inverter station showed high relevance in resource-related and selected toxicity-related impacts. This confirms that the environmental assessment of photovoltaic infrastructure should include both modules and balance-of-system components.

Recycling-oriented material recovery reduced selected environmental burdens and, in several categories, generated environmental credits associated with the avoided production of primary materials. This demonstrates that post-consumer material management is a decisive factor in the life cycle performance of photovoltaic power plants and should be integrated with material selection, component design, dismantling procedures, and recovery-oriented planning. However, these benefits are conditional on high recovery efficiency, sufficient secondary material quality, controlled treatment of complex fractions, and the actual substitution of primary materials by recovered materials.

Under the adopted modelling assumptions, the prospective perovskite module scenario showed lower environmental burdens than the mono-crystalline silicon configuration. However, this result should be treated as a scenario-based indication of potential environmental benefits rather than as a confirmed industrial-scale advantage because perovskite photovoltaic technology is still developing. In particular, this conclusion is conditional on effective encapsulation during operation and controlled end-of-life management of lead-containing fractions because the uncontrolled release of lead could increase toxicity-related impacts and reduce the environmental advantage of the PSC scenario.

These comparative conclusions should be interpreted together with the key modelling assumptions, especially module service life and degradation, annual electricity output, electricity mix, manufacturing energy demand, recycling efficiency, secondary material quality, and recovery-related substitution ratios.

The IPCC-based greenhouse gas assessment confirmed that recycling-oriented material recovery may reduce the carbon footprint of both analysed photovoltaic material configurations. At the same time, the ReCiPe 2016 results showed that climate-related indicators should be interpreted together with broader impact categories related to human health, ecosystem quality, toxicity, water consumption, and resource scarcity.

The study confirms that sustainable photovoltaic infrastructure requires a material-oriented and circular approach. Improving the environmental performance of photovoltaic power plants not only depends on generating low-carbon electricity but also on reducing material-related burdens, increasing recovery potential, and developing effective post-consumer material management pathways after the end of the technical facility’s life.

## Figures and Tables

**Table 1:** Material recovery assumptions used in the recycling-oriented material recovery pathway.

Material or Component Fraction	Main Source in the Analysed System	Assumed Recycling or Recovery Process	Recovery Rate Assumed in the Model	Substitution and Credit Assumption
Aluminium frames and support elements	PV module frames and selected structural elements	Mechanical dismantling, sorting, and remelting as secondary aluminium	95%	Recovered aluminium substitutes primary aluminium only for the quality-compliant recovered fraction
Steel support structures and container fractions	Support structures, inverter station container, and transformer-related steel elements	Mechanical dismantling, magnetic separation, and steel scrap recycling	90%	Recovered steel substitutes primary steel for the recovered fraction
Copper cables and conductive elements	Electrical installation, cabling, transformer and inverter-related conductive parts	Cable separation, shredding, sorting, and copper recovery	95%	Recovered copper substitutes for primary copper in the recovered fraction
Solar glass	PV module glass layers	Module dismantling, glass separation, cleaning, and cullet recovery	80%	Recovered glass substitutes for primary glass raw material or glass cullet demand, where quality requirements are met
Silicon or semiconductor-rich fraction in sc-Si modules	Mono-crystalline silicon PV cells and related module layers	Mechanical and thermal separation followed by recovery of silicon-rich and metal-containing fractions	80%	Recovered silicon-rich fraction only receives avoided-production credits for the recoverable and reusable fraction
Semiconductor-rich and lead-containing fraction in PSC modules	Perovskite absorber layer and associated coated-glass or semiconductor fractions	Controlled separation, recovery, or stabilisation of semiconductor-rich and lead-containing fractions	70%	Credits are only assigned to recoverable fractions managed in controlled treatment systems. Non-recovered lead-containing residues are treated as controlled residual streams
Recoverable metals from inverter and transformer equipment	Inverter station, transformer, switchgear, and auxiliary electrical equipment	Dismantling and recovery of metal-rich fractions from electrical and electronic equipment	70%	Recovered metals substitute for corresponding primary metals according to the recoverable fraction
Polymeric and mixed residues	Encapsulants, backsheets, insulation, mixed plastics, and composite residues	Mechanical separation or residual treatment, depending on separability	0–20%	Avoided-production credits are assigned only to separable recyclable fractions. Mixed residues receive no material recovery credit

**Table 2:** ReCiPe 2016 characterisation results for the mono-crystalline silicon photovoltaic power plant by component and post-consumer material management pathway (part 1/2).

No	Component of Photovoltaic Power Plant	Support Structure		Photovoltaic Panels		Unit
	Post-Consumer Material Management Pathway	Landfill	Recycling	Landfill	Recycling	
	Impact Category					
1	Global warming, human health	1.26 × 10^−1^	8.92 × 10^−2^	7.44 × 10^−1^	−9.37 × 10^−2^	DALY
2	Stratospheric ozone depletion	1.01 × 10^−5^	8.81 × 10^−6^	1.43 × 10^−4^	−3.12 × 10^−6^	DALY
3	Ionising radiation	1.62 × 10^−5^	1.33 × 10^−5^	1.43 × 10^−3^	−5.00 × 10^−4^	DALY
4	Ozone formation, human health	2.25 × 10^−4^	2.22 × 10^−4^	1.45 × 10^−3^	8.44 × 10^−5^	DALY
5	Fine particulate matter formation	1.05 × 10^−1^	1.05 × 10^−1^	7.93 × 10^−1^	−5.43 × 10^−2^	DALY
6	Human carcinogenic toxicity	1.62 × 10^−1^	1.59 × 10^−1^	3.91 × 10^−1^	−1.16 × 10^−2^	DALY
7	Human non-carcinogenic toxicity	1.29 × 10^−1^	1.59 × 10^−2^	5.97 × 10^−1^	5.14 × 10^−1^	DALY
8	Water consumption, human health	1.59 × 10^−2^	2.03 × 10^−3^	3.62 × 10^1^	−1.66 × 10^1^	DALY
9	Global warming, terrestrial ecosystems	3.79 × 10^−4^	2.69 × 10^−4^	2.25 × 10^−3^	−2.83 × 10^−4^	species/year
10	Global warming, freshwater ecosystems	1.03 × 10^−8^	7.35 × 10^−9^	6.13 × 10^−8^	−7.73 × 10^−9^	species/year
11	Ozone formation, terrestrial ecosystems	3.44 × 10^−5^	3.39 × 10^−5^	2.09 × 10^−4^	1.24 × 10^−5^	species/year
12	Terrestrial acidification	5.27 × 10^−5^	5.19 × 10^−5^	6.79 × 10^−4^	−3.88 × 10^−5^	species/year
13	Freshwater eutrophication	1.10 × 10^−4^	3.24 × 10^−5^	2.88 × 10^−4^	2.09 × 10^−4^	species/year
14	Marine eutrophication	7.96 × 10^−8^	4.46 × 10^−9^	1.34 × 10^−7^	1.29 × 10^−7^	species/year
15	Terrestrial ecotoxicity	1.75 × 10^−6^	1.74 × 10^−6^	3.30 × 10^−5^	1.60 × 10^−5^	species/year
16	Freshwater ecotoxicity	1.99 × 10^−5^	4.01 × 10^−6^	9.32 × 10^−5^	8.57 × 10^−5^	species/year
17	Marine ecotoxicity	4.03 × 10^−6^	8.58 × 10^−7^	2.12 × 10^−5^	1.95 × 10^−5^	species/year
18	Land use	1.49 × 10^−5^	1.40 × 10^−5^	1.22 × 10^−4^	4.54 × 10^−5^	species/year
19	Water consumption, terrestrial ecosystems	9.76 × 10^−5^	1.30 × 10^−5^	2.20 × 10^−1^	−1.01 × 10^−1^	species/year
20	Water consumption, aquatic ecosystems	4.78 × 10^−9^	9.95 × 10^−10^	9.85 × 10^−6^	−4.50 × 10^−6^	species/year
21	Mineral resource scarcity	7.97 × 10^2^	7.96 × 10^2^	6.91 × 10^3^	3.84 × 10^2^	USD
22	Fossil resource scarcity	3.58 × 10^3^	3.52 × 10^3^	5.13 × 10^4^	−3.71 × 10^3^	USD

**Table 3:** ReCiPe 2016 characterisation results for the mono-crystalline silicon photovoltaic power plant by component and post-consumer material management pathway (part 2/2).

No	Component of Photovoltaic Power Plant	Inverter Station		Electrical Installation		Unit
	Post-Consumer Material Management Pathway	Landfill	Recycling	Landfill	Recycling	
	Impact Category					
1	Global warming, human health	6.16 × 10^−1^	5.01 × 10^−1^	2.19 × 10^−2^	1.38 × 10^−4^	DALY
2	Stratospheric ozone depletion	2.88 × 10^−4^	2.66 × 10^−4^	2.27 × 10^−5^	1.43 × 10^−7^	DALY
3	Ionising radiation	1.66 × 10^−3^	1.37 × 10^−3^	2.82 × 10^−5^	1.77 × 10^−7^	DALY
4	Ozone formation, human health	1.78 × 10^−3^	1.58 × 10^−3^	1.50 × 10^−4^	9.43 × 10^−7^	DALY
5	Fine particulate matter formation	1.08 × 10^0^	9.54 × 10^−1^	1.95 × 10^−1^	1.23 × 10^−3^	DALY
6	Human carcinogenic toxicity	4.28 × 10^−1^	3.53 × 10^−1^	1.47 × 10^−2^	9.25 × 10^−5^	DALY
7	Human non-carcinogenic toxicity	1.73 × 10^0^	1.70 × 10^0^	1.59 × 10^−1^	1.00 × 10^−3^	DALY
8	Water consumption, human health	5.70 × 10^0^	−2.22 × 10^0^	5.19 × 10^−1^	3.26 × 10^−3^	DALY
9	Global warming, terrestrial ecosystems	1.86 × 10^−3^	1.51 × 10^−3^	6.59 × 10^−5^	4.14 × 10^−7^	species/year
10	Global warming, freshwater ecosystems	5.07 × 10^−8^	4.13 × 10^−8^	1.80 × 10^−9^	1.13 × 10^−11^	species/year
11	Ozone formation, terrestrial ecosystems	2.58 × 10^−4^	2.28 × 10^−4^	2.16 × 10^−5^	1.36 × 10^−7^	species/year
12	Terrestrial acidification	9.98 × 10^−4^	8.90 × 10^−4^	1.34 × 10^−4^	8.43 × 10^−7^	species/year
13	Freshwater eutrophication	3.24 × 10^−4^	3.11 × 10^−4^	2.48 × 10^−5^	1.56 × 10^−7^	species/year
14	Marine eutrophication	1.84 × 10^−7^	1.61 × 10^−7^	1.11 × 10^−8^	6.98 × 10^−11^	species/year
15	Terrestrial ecotoxicity	3.15 × 10^−4^	3.12 × 10^−4^	6.66 × 10^−5^	4.19 × 10^−7^	species/year
16	Freshwater ecotoxicity	1.56 × 10^−4^	1.52 × 10^−4^	8.66 × 10^−6^	5.45 × 10^−8^	species/year
17	Marine ecotoxicity	3.25 × 10^−5^	3.17 × 10^−5^	1.94 × 10^−6^	1.22 × 10^−8^	species/year
18	Land use	2.20 × 10^−4^	2.09 × 10^−4^	2.85 × 10^−5^	1.79 × 10^−7^	species/year
19	Water consumption, terrestrial ecosystems	3.47 × 10^−2^	−1.35 × 10^−2^	3.16 × 10^−3^	1.99 × 10^−5^	species/year
20	Water consumption, aquatic ecosystems	1.56 × 10^−6^	−6.00 × 10^−7^	1.41 × 10^−7^	8.87 × 10^−10^	species/year
21	Mineral resource scarcity	4.50 × 10^4^	4.41 × 10^4^	2.79 × 10^3^	1.75 × 10^1^	USD
22	Fossil resource scarcity	6.52 × 10^4^	5.69 × 10^4^	1.48 × 10^3^	9.31 × 10^0^	USD

**Table 4:** ReCiPe 2016 characterisation results for the prospective perovskite module scenario by component and post-consumer material management pathway (part 1/2).

No	Component of Photovoltaic Power Plant	Support Structure		Photovoltaic Panels		Unit
	Post-Consumer Material Management Pathway	Landfill	Recycling	Landfill	Recycling	
	Impact Category					
1	Global warming, human health	1.14 × 10^−1^	8.07 × 10^−2^	6.73 × 10^−1^	−8.48 × 10^−2^	DALY
2	Stratospheric ozone depletion	9.14 × 10^−6^	7.97 × 10^−6^	1.29 × 10^−4^	−2.82 × 10^−6^	DALY
3	Ionising radiation	1.46 × 10^−5^	1.20 × 10^−5^	1.29 × 10^−3^	−4.52 × 10^−4^	DALY
4	Ozone formation, human health	2.03 × 10^−4^	2.01 × 10^−4^	1.31 × 10^−3^	7.63 × 10^−5^	DALY
5	Fine particulate matter formation	9.50 × 10^−2^	9.49 × 10^−2^	7.17 × 10^−1^	−4.91 × 10^−2^	DALY
6	Human carcinogenic toxicity	1.47 × 10^−1^	1.44 × 10^−1^	3.54 × 10^−1^	−1.05 × 10^−2^	DALY
7	Human non-carcinogenic toxicity	1.17 × 10^−1^	1.44 × 10^−2^	5.10 × 10^−1^	3.65 × 10^−1^	DALY
8	Water consumption, human health	1.44 × 10^−2^	1.84 × 10^−3^	3.27 × 10^1^	−1.50 × 10^1^	DALY
9	Global warming, terrestrial ecosystems	3.43 × 10^−4^	2.03 × 10^−4^	2.03 × 10^−3^	−2.06 × 10^−4^	species/year
10	Global warming, freshwater ecosystems	9.32 × 10^−9^	6.65 × 10^−9^	5.55 × 10^−8^	−6.99 × 10^−9^	species/year
11	Ozone formation, terrestrial ecosystems	3.11 × 10^−5^	3.07 × 10^−5^	1.89 × 10^−4^	1.12 × 10^−5^	species/year
12	Terrestrial acidification	4.77 × 10^−5^	4.69 × 10^−5^	6.14 × 10^−4^	−3.51 × 10^−5^	species/year
13	Freshwater eutrophication	9.95 × 10^−5^	2.93 × 10^−5^	2.61 × 10^−4^	1.89 × 10^−4^	species/year
14	Marine eutrophication	7.20 × 10^−8^	4.03 × 10^−9^	1.21 × 10^−7^	1.17 × 10^−7^	species/year
15	Terrestrial ecotoxicity	1.58 × 10^−6^	1.57 × 10^−6^	2.99 × 10^−5^	1.45 × 10^−5^	species/year
16	Freshwater ecotoxicity	1.80 × 10^−5^	3.63 × 10^−6^	8.43 × 10^−5^	7.75 × 10^−5^	species/year
17	Marine ecotoxicity	3.64 × 10^−6^	7.76 × 10^−7^	1.92 × 10^−5^	1.76 × 10^−5^	species/year
18	Land use	1.35 × 10^−5^	1.27 × 10^−5^	1.10 × 10^−4^	4.11 × 10^−5^	species/year
19	Water consumption, terrestrial ecosystems	8.83 × 10^−5^	1.18 × 10^−5^	1.99 × 10^−1^	−9.13 × 10^−2^	species/year
20	Water consumption, aquatic ecosystems	4.32 × 10^−9^	9.00 × 10^−10^	8.91 × 10^−6^	−4.07 × 10^−6^	species/year
21	Mineral resource scarcity	7.21 × 10^2^	7.20 × 10^2^	6.25 × 10^3^	3.47 × 10^2^	USD
22	Fossil resource scarcity	3.24 × 10^3^	2.18 × 10^3^	4.64 × 10^4^	−2.35 × 10^3^	USD

**Table 5:** ReCiPe 2016 characterisation results for the prospective perovskite module scenario by component and post-consumer material management pathway (part 2/2).

No	Component of Photovoltaic Power Plant	Inverter Station		Electrical Installation		Unit
	Post-Consumer Material Management Pathway	Landfill	Recycling	Landfill	Recycling	
	Impact Category					
1	Global warming, human health	5.57 × 10^−1^	4.53 × 10^−1^	1.98 × 10^−2^	1.25 × 10^−4^	DALY
2	Stratospheric ozone depletion	2.60 × 10^−4^	2.41 × 10^−4^	2.05 × 10^−5^	1.29 × 10^−7^	DALY
3	Ionising radiation	1.50 × 10^−3^	1.24 × 10^−3^	2.55 × 10^−5^	1.60 × 10^−7^	DALY
4	Ozone formation, human health	1.61 × 10^−3^	1.43 × 10^−3^	1.36 × 10^−4^	8.54 × 10^−7^	DALY
5	Fine particulate matter formation	9.77 × 10^−1^	8.62 × 10^−1^	1.76 × 10^−1^	1.11 × 10^−3^	DALY
6	Human carcinogenic toxicity	3.87 × 10^−1^	3.19 × 10^−1^	1.33 × 10^−2^	8.36 × 10^−5^	DALY
7	Human non-carcinogenic toxicity	1.57 × 10^0^	1.46 × 10^0^	1.44 × 10^−1^	9.05 × 10^−4^	DALY
8	Water consumption, human health	5.16 × 10^0^	−2.01 × 10^0^	4.70 × 10^−1^	2.95 × 10^−3^	DALY
9	Global warming, terrestrial ecosystems	1.68 × 10^−3^	1.17 × 10^−3^	5.96 × 10^−5^	3.75 × 10^−7^	species/year
10	Global warming, freshwater ecosystems	4.59 × 10^−8^	3.74 × 10^−8^	1.63 × 10^−9^	1.02 × 10^−11^	species/year
11	Ozone formation, terrestrial ecosystems	2.33 × 10^−4^	2.06 × 10^−4^	1.95 × 10^−5^	1.23 × 10^−7^	species/year
12	Terrestrial acidification	9.03 × 10^−4^	8.05 × 10^−4^	1.21 × 10^−4^	7.62 × 10^−7^	species/year
13	Freshwater eutrophication	2.93 × 10^−4^	2.81 × 10^−4^	2.24 × 10^−5^	1.41 × 10^−7^	species/year
14	Marine eutrophication	1.66 × 10^−7^	1.46 × 10^−7^	1.00 × 10^−8^	6.32 × 10^−11^	species/year
15	Terrestrial ecotoxicity	2.85 × 10^−4^	2.82 × 10^−4^	6.02 × 10^−5^	3.79 × 10^−7^	species/year
16	Freshwater ecotoxicity	1.41 × 10^−4^	1.37 × 10^−4^	7.83 × 10^−6^	4.93 × 10^−8^	species/year
17	Marine ecotoxicity	2.94 × 10^−5^	2.87 × 10^−5^	1.76 × 10^−6^	1.10 × 10^−8^	species/year
18	Land use	1.99 × 10^−4^	1.89 × 10^−4^	2.58 × 10^−5^	1.62 × 10^−7^	species/year
19	Water consumption, terrestrial ecosystems	3.14 × 10^−2^	−1.22 × 10^−2^	2.86 × 10^−3^	1.80 × 10^−5^	species/year
20	Water consumption, aquatic ecosystems	1.41 × 10^−6^	−5.43 × 10^−7^	1.28 × 10^−7^	8.02 × 10^−10^	species/year
21	Mineral resource scarcity	4.07 × 10^4^	3.99 × 10^4^	2.52 × 10^3^	1.59 × 10^1^	USD
22	Fossil resource scarcity	5.90 × 10^4^	4.75 × 10^4^	1.34 × 10^3^	8.42 × 10^0^	USD

**Table 6:** Component-level ReCiPe 2016 results for the water consumption, human health category, by photovoltaic material configuration and post-consumer material management pathway.

Component of Photovoltaic Power Plant	Support Structure		Photovoltaic Panels		Inverter Station		Electrical Installation		Unit
Post-Consumer Material Management Pathway	Landfill	Recycling	Landfill	Recycling	Landfill	Recycling	Landfill	Recycling	
Photovoltaic Module Technology									
sc-Si	×	×	3.60 × 10^1^	−1.70 × 10^1^	6.00 × 10^0^	−2.00 × 10^0^	1.00 × 10^0^	×	DALY
PSC	×	×	3.30 × 10^1^	−1.50 × 10^1^	5.00 × 10^0^	−2.00 × 10^0^	×	×	

**Table 7:** Component-level ReCiPe 2016 results for the human non-carcinogenic toxicity category by photovoltaic material configuration and post-consumer material management pathway.

Component of Photovoltaic Power Plant	Support Structure		Photovoltaic Panels		Inverter Station		Electrical Installation		Unit
Post-Consumer Material Management Pathway	Landfill	Recycling	Landfill	Recycling	Landfill	Recycling	Landfill	Recycling	
Photovoltaic Module Technology									
sc-Si	1.00 × 10^−1^	×	6.00 × 10^−1^	5.00 × 10^−1^	1.70 × 10^0^	1.70 × 10^0^	2.00 × 10^−1^	×	DALY
PSC	1.00 × 10^−1^	×	5.00 × 10^−1^	4.00 × 10^−1^	1.60 × 10^0^	1.50 × 10^0^	1.00 × 10^−1^	×	

**Table 8:** Component-level ReCiPe 2016 results for the water consumption, terrestrial ecosystem category, by photovoltaic material configuration and post-consumer material management pathway.

Component of Photovoltaic Power Plant	Support Structure		Photovoltaic Panels		Inverter Station		Electrical Installation		Unit
Post-Consumer Material Management Pathway	Landfill	Recycling	Landfill	Recycling	Landfill	Recycling	Landfill	Recycling	
Photovoltaic Module Technology									
sc-Si	×	×	2.20 × 10^−1^	−1.00 × 10^−1^	3.00 × 10^−2^	−1.00 × 10^−2^	×	×	species/year
PSC	×	×	2.00 × 10^−1^	−9.00 × 10^−2^	3.00 × 10^−2^	−1.00 × 10^−2^	×	×	

**Table 9:** Component-level ReCiPe 2016 results for the global warming, terrestrial ecosystems category, by photovoltaic material configuration and post-consumer material management pathway.

Component of Photovoltaic Power Plant	Support Structure		Photovoltaic Panels		Inverter Station		Electrical Installation		Unit
Post-Consumer Material Management Pathway	Landfill	Recycling	Landfill	Recycling	Landfill	Recycling	Landfill	Recycling	
Photovoltaic Module Technology									
sc-Si	4.00 × 10^−4^	3.00 × 10^−4^	2.30 × 10^−3^	−3.00 × 10^−4^	1.90 × 10^−3^	1.50 × 10^−3^	1.00 × 10^−4^	×	species/year
PSC	3.00 × 10^−4^	2.00 × 10^−4^	2.00 × 10^−3^	−2.00 × 10^−4^	1.70 × 10^−4^	1.20 × 10^−3^	1.00 × 10^−4^	×	

**Table 10:** Endpoint ReCiPe 2016 results for the analysed photovoltaic material configurations by area of protection, component, and post-consumer material management pathway.

Component of Photovoltaic Power Plant		Support Structure		Photovoltaic Panels		Inverter Station		Electrical Installation		Unit
Post-Consumer Material Management Pathway		Landfill	Recycling	Landfill	Recycling	Landfill	Recycling	Landfill	Recycling	
Area of Influence	Photovoltaic Module Technology									
Human health	sc-Si	5.38 × 10^−1^	3.71 × 10^−1^	3.87 × 10^1^	−1.62 × 10^1^	9.56 × 10^0^	1.29 × 10^0^	9.10 × 10^−1^	5.72 × 10^−3^	DALY
	PSC	4.87 × 10^−1^	3.36 × 10^−1^	3.50 × 10^1^	−1.48 × 10^1^	8.65 × 10^0^	1.09 × 10^0^	8.23 × 10^−1^	5.18 × 10^−3^	
Ecosystems	sc-Si	7.14 × 10^−4^	4.21 × 10^−4^	2.24 × 10^−1^	−1.01 × 10^−1^	3.89 × 10^−2^	−9.86 × 10^−3^	3.51 × 10^−3^	2.21 × 10^−5^	species/year
	PSC	6.46 × 10^−4^	3.41 × 10^−4^	2.02 × 10^−1^	−9.12 × 10^−2^	3.52 × 10^−2^	−9.12 × 10^−3^	3.18 × 10^−3^	2.00 × 10^−5^	
Resources	sc-Si	4.38 × 10^3^	4.32 × 10^3^	5.82 × 10^4^	−3.33 × 10^3^	1.10 × 10^5^	1.01 × 10^5^	4.27 × 10^3^	2.69 × 10^1^	USD
	PSC	3.96 × 10^3^	2.90 × 10^3^	5.26 × 10^4^	−2.01 × 10^3^	9.97 × 10^4^	8.74 × 10^4^	3.86 × 10^3^	2.43 × 10^1^	

**Table 11:** Endpoint ReCiPe 2016 results for the human health area by photovoltaic material configuration, component, and post-consumer material management pathway.

Component of Photovoltaic Power Plant	Support Structure		Photovoltaic Panels		Inverter Station		Electrical Installation		Unit
Post-Consumer Material Management Pathway	Landfill	Recycling	Landfill	Recycling	Landfill	Recycling	Landfill	Recycling	
Photovoltaic Module Technology									
sc-Si	1.00 × 10^0^	×	3.90 × 10^1^	−1.60 × 10^1^	1.00 × 10^1^	1.00 × 10^0^	1.00 × 10^0^	×	DALY
PSC	×	×	3.50 × 10^1^	−1.50 × 10^1^	9.00 × 10^0^	1.00 × 10^0^	1.00 × 10^0^	×	

**Table 12:** Endpoint ReCiPe 2016 results for the ecosystems area by photovoltaic material configuration, component, and post-consumer material management pathway.

Component of Photovoltaic Power Plant	Support Structure		Photovoltaic Panels		Inverter Station		Electrical Installation		Unit
Post-Consumer Material Management Pathway	Landfill	Recycling	Landfill	Recycling	Landfill	Recycling	Landfill	Recycling	
Photovoltaic Module Technology									
sc-Si	×	×	2.20 × 10^−1^	−1.00 × 10^−2^	4.00 × 10^−2^	−1.00 × 10^−2^	×	×	species/year
PSC	×	×	2.00 × 10^−2^	−9.00 × 10^−2^	4.00 × 10^−2^	−1.00 × 10^−2^	×	×	

**Table 13:** Endpoint ReCiPe 2016 results for the resources area by photovoltaic material configuration, component, and post-consumer material management pathway.

Component of Photovoltaic Power Plant	Support Structure		Photovoltaic Panels		Inverter Station		Electrical Installation		Unit
Post-Consumer Material Management Pathway	Landfill	Recycling	Landfill	Recycling	Landfill	Recycling	Landfill	Recycling	
Photovoltaic Module Technology									
sc-Si	4.37 × 10^3^	4.31 × 10^3^	5.82 × 10^4^	−3.32 × 10^3^	1.10 × 10^5^	1.01 × 10^5^	4.27 × 10^3^	2.70 × 10^1^	USD
PSC	3.96 × 10^3^	2.90 × 10^3^	5.26 × 10^4^	−2.00 × 10^3^	9.96 × 10^4^	8.73 × 10^4^	3.86 × 10^3^	2.40 × 10^1^	

**Table 14:** Weighted ReCiPe 2016 results for the analysed photovoltaic material configurations by area of protection, component, and post-consumer material management pathway.

Component of Photovoltaic Power Plant		Support Structure		Photovoltaic Panels		Inverter Station		Electrical Installation		Unit
Post-Consumer Material Management Pathway		Landfill	Recycling	Landfill	Recycling	Landfill	Recycling	Landfill	Recycling	
Area of influence	Photovoltaic Module Technology									
Human health	sc-Si	8.97 × 10^3^	6.19 × 10^3^	6.46 × 10^5^	−2.72 × 10^5^	1.59 × 10^5^	2.69 × 10^4^	1.52 × 10^4^	9.56 × 10^1^	Pt
	PSC	8.95 × 10^3^	1.90 × 10^4^	3.38 × 10^5^	−1.39 × 10^5^	1.02 × 10^5^	1.09 × 10^4^	1.95 × 10^4^	1.49 × 10^2^	
Ecosystems	sc-Si	1.93 × 10^2^	1.14 × 10^2^	6.06 × 10^4^	−2.72 × 10^4^	1.05 × 10^4^	−2.66 × 10^3^	9.49 × 10^2^	5.97 × 10^0^	
	PSC	1.57 × 10^2^	9.28 × 10^1^	2.50 × 10^4^	−1.05 × 10^4^	9.50 × 10^3^	−2.41 × 10^3^	7.56 × 10^2^	4.75 × 10^0^	
Resources	sc-Si	3.13 × 10^1^	3.08 × 10^1^	4.16 × 10^2^	−2.38 × 10^1^	7.87 × 10^2^	7.22 × 10^2^	3.05 × 10^1^	1.92 × 10^−1^	
	PSC	−1.62 × 10^3^	−1.40 × 10^4^	−1.01 × 10^4^	−1.88 × 10^4^	4.30 × 10^4^	1.41 × 10^4^	−7.37 × 10^3^	−7.31 × 10^1^	
Total	sc-Si	9.19 × 10^3^	6.33 × 10^3^	7.07 × 10^5^	−2.99 × 10^5^	1.71 × 10^5^	2.50 × 10^4^	1.62 × 10^4^	1.02 × 10^2^	
	PSC	7.49 × 10^3^	5.16 × 10^3^	3.53 × 10^5^	−1.69 × 10^5^	1.54 × 10^5^	2.26 × 10^4^	1.29 × 10^4^	8.10 × 10^1^	

**Table 15:** Total weighted ReCiPe 2016 results by photovoltaic material configuration, component, and post-consumer material management pathway.

Component of Photovoltaic Power Plant	Support Structure		Photovoltaic Panels		Inverter Station		Electrical Installation		Unit
Post-Consumer Material Management Pathway	Landfill	Recycling	Landfill	Recycling	Landfill	Recycling	Landfill	Recycling	
Photovoltaic Module Technology									
sc-Si	9.19 × 10^3^	6.33 × 10^3^	7.07 × 10^5^	−2.99 × 10^5^	1.70 × 10^5^	2.49 × 10^4^	1.61 × 10^4^	1.02 × 10^2^	Pt
PSC	7.48 × 10^3^	5.15 × 10^3^	3.52 × 10^5^	−1.68 × 10^5^	1.54 × 10^5^	2.25 × 10^4^	1.28 × 10^4^	8.10 × 10^1^	

**Table 16:** Whole-system total weighted ReCiPe 2016 results by photovoltaic material configuration and post-consumer material management pathway.

Post-Consumer Material Management Pathway	Landfill	Recycling	Unit
Photovoltaic Module Technology			
sc-Si	9.03 × 10^5^	−2.67 × 10^5^	Pt
PSC	5.27 × 10^5^	−1.40 × 10^5^	

**Table 17:** Component-level IPCC GWP100 results by greenhouse gas emission group, photovoltaic material configuration, and post-consumer material management pathway.

Component of Photovoltaic Power Plant		Support Structure		Photovoltaic Panels		Inverter Station		Electrical Installation		Unit
Post-Consumer Material Management Pathway		Landfill	Recycling	Landfill	Recycling	Landfill	Recycling	Landfill	Recycling	
Impact Category	Photovoltaic Module Technology									
GWP100–fossil	sc-Si	9.63 × 10^4^	9.41 × 10^4^	7.34 × 10^5^	−1.62 × 10^5^	6.37 × 10^5^	5.03 × 10^5^	1.90 × 10^4^	1.00 × 10^0^	kg CO_2_ eq
	PSC	7.84 × 10^4^	3.67 × 10^4^	3.97 × 10^5^	−7.91 × 10^4^	5.76 × 10^5^	4.55 × 10^5^	1.51 × 10^4^	1.00 × 10^0^	
GWP100–biogenic	sc-Si	2.99 × 10^4^	3.90 × 10^1^	4.92 × 10^4^	4.91 × 10^4^	1.95 × 10^4^	1.02 × 10^4^	3.41 × 10^3^	×	
	PSC	2.43 × 10^4^	2.40 × 10^1^	2.76 × 10^4^	2.42 × 10^4^	1.76 × 10^4^	9.22 × 10^3^	2.71 × 10^3^	×	
GWP100–land transformation	sc-Si	6.40 × 10^1^	6.40 × 10^1^	1.54 × 10^2^	1.54 × 10^2^	3.43 × 10^3^	3.34 × 10^4^	1.30 × 10^1^	×	
	PSC	5.20 × 10^1^	4.10 × 10^1^	8.10 × 10^1^	7.30 × 10^1^	3.10 × 10^3^	3.02 × 10^3^	1.00 × 10^1^	×	

**Table 18:** Summary of component-level IPCC greenhouse gas emissions by photovoltaic material configuration and post-consumer material management pathway.

Component of Photovoltaic Power Plant	Support Structure		Photovoltaic Panels		Inverter Station		Electrical Installation		Unit
Post-Consumer Material Management Pathway	Landfill	Recycling	Landfill	Recycling	Landfill	Recycling	Landfill	Recycling	
Photovoltaic Module Technology									
sc-Si	1.26 × 10^5^	4.54 × 10^5^	7.83 × 10^5^	−1.12 × 10^5^	6.59 × 10^5^	5.16 × 10^5^	2.24 × 10^4^	1.00 × 10^0^	kg CO_2_ eq
PSC	1.02 × 10^5^	3.07 × 10^5^	4.24 × 10^5^	−7.48 × 10^4^	5.47 × 10^5^	3.07 × 10^5^	1.78 × 10^4^	1.00 × 10^0^	

**Table 19:** Whole-system IPCC greenhouse gas emissions by photovoltaic material configuration and post-consumer material management pathway.

Post-Consumer Material Management Pathway	Landfill	Recycling	Unit
Photovoltaic Module Technology			
sc-Si	1.59 × 10^6^	8.57 × 10^5^	kg CO_2_ eq
PSC	9.89 × 10^5^	5.40 × 10^5^	

## Data Availability

The original contributions presented in the study are included in the article. Further inquiries can be directed to the corresponding author.

## Abbreviations

The following abbreviations are used in this manuscript:

CED	Cumulative Energy Demand
CO_2_ eq	Carbon Dioxide Equivalent
DALY	Disability-Adjusted Life Year
GWP	Global Warming Potential
IPCC	Intergovernmental Panel on Climate Change
LCA	Life Cycle Assessment
LCI	Life Cycle Inventory
LCIA	Life Cycle Impact Assessment
PV	Photovoltaic
PSC	Perovskite Solar Cell
Pt	Environmental Point
sc-Si	Mono-Crystalline Silicon
USD	United States Dollar
